# Mitigating Strain Localization via Stabilized Phase Boundaries for Strengthening Multi‐Principal Element Alloys

**DOI:** 10.1002/advs.202414783

**Published:** 2025-03-08

**Authors:** Jinliang Du, Shukuan Guo, Hangqi Feng, Weijie Li, Zhixin Huang, Zhongji Sun, Yunli Feng, Pei Wang, Ying Li

**Affiliations:** ^1^ School of Naval Architecture Ocean and Energy Power Engineering Wuhan University of Technology Wuhan 430063 P. R. China; ^2^ Beijing Institute of Technology Beijing Institute of Technology Zhuhai 519088 P. R. China; ^3^ Institute of Materials Research and Engineering (IMRE) Agency for Science, Technology and Research (A*STAR) Singapore 138634 Republic of Singapore; ^4^ State Key Laboratory of High‐Performance Ceramics and Superfine Microstructure, Shanghai Institute of Ceramics Chinese Academy of Sciences Shanghai 201899 P. R. China; ^5^ Key Laboratory of Modern Metallurgical Technology of Ministry of Education North China University of Science and Technology Tangshan 063210 P. R. China; ^6^ Engineering Cluster Singapore Institute of Technology Singapore 519961 Republic of Singapore

**Keywords:** bionic materials, microstructure design, multi‐principal element alloys, phase boundaries stability, strain distribution

## Abstract

Multi‐principal element alloys (MPEA) demonstrate exceptional stability during rapid solidification, making them ideal candidates for additive manufacturing and other high‐design‐flexibility techniques. Unexpectedly, MPEA failure often mimics that of conventional metals, with strain localization along phase or grain boundaries leading to typical crack initiation. Most strategies aim at reducing strain localization either suppress the formation of high‐energy sites or dissipate energy at crack tips to enhance toughness, rarely achieving a synergy of both. Inspired by the microstructure of mouse enamel, nanoscale body‐centered cubic (BCC) and face‐centered cubic (FCC) phases into MPEAs are introduced, stabilized at phase boundaries to provide ample plastic space for dislocation‐mediated deformation. This approach overcomes the local hardening limitations of nanoscale alloys and harmonizes traditional toughening mechanisms—such as crack deflection, blocking, and bridging—to mitigate strain localization. These mechanisms impart the alloy with ultra‐high tensile strength (≈1458.1 MPa) and ductility (≈21.2%) without requiring heat treatment. Atomic calculations reveal that partial atomic plane migration drives continuous dislocation transfer across phases. This study uncovers fundamental but latent mechanical mechanisms in MPEAs, advancing understanding of ultra‐strong bioinspired alloys.

## Graphical abstract

Strain localization along phase or grain boundaries leads to crack initiation typical of alloys. In multi‐principal element alloys, the enamel‐like microstructure is stabilized by phase boundaries. This facilitates trans‐phase migration of semi‐atomic surfaces and provides sufficient plastic space for dislocation‐mediated deformation. This approach overcomes the limitations of localized hardening of nanoscale microstructure alloys to mitigate strain localization.

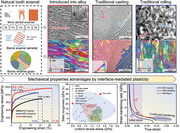

## 1 Introduction

Unintentional macro/micro defects in engineering structural materials can lead to premature or excessive local strain concentrations, causing catastrophic equipment failure.^[^ ^1^, ^2^, ^3^ ^]^ Strain concentration is a typical mechanical behavior where structural materials exhibit abnormal mechanical behavior at local positions under stress. This often severely undermines the overall load‐bearing capacity of the material, causing and accelerating issues such as crack initiation, embrittlement, and fracture, hereby threatening the service life of the material.^[^ ^4^ ^]^ This phenomenon commonly occurs in various structural metal materials, especially ultra‐high‐strength ultrafine/nano grain structural alloys.^[^ ^5^, ^6^ ^]^

From a mechanical perspective, the ideal strain concentration resistance strategy is to suppress the generation of strain concentration sites and dissipate the strain energy at the crack tip. However, the microstructures that affect these two stages seem to be in a contradictory state. More specifically, in NG/UFG high‐strength alloys, the limited diffusion and interaction of dislocations within a small volume inhibit the continuous nucleation of new dislocations and the sustained activation of displacement deformation mechanisms.^[^ ^7^, ^8^ ^]^ Under external load, deformation is regulated by grain boundary sliding and separation to accommodate the mutual compression of adjacent fine grains.^[^ ^9^ ^]^ Dislocation mechanism‐driven intragranular plasticity becomes more difficult, but this leads to a small space passivation crack propagation mechanism, and this strength enhancement is usually due to geometric space constraints at the expense of macro/micro local damage resistance and plastic stability.

Currently, applying thermal or pressure fields—such as annealing, hot isostatic pressing, or high‐temperature and high‐pressure treatments—can mitigate potential internal strain concentration sites. However, these treatments often trigger grain recovery and recrystallization (or growth), tending to degrade the originally designed microstructural configuration. Such processes may also reduce strength or promote the precipitation and growth of secondary phase particles at grain boundaries and other high‐energy sites, creating new local strain concentrations and potentially accelerating brittleness. This undermines the alloy's capacity to dissipate energy. Therefore, it is essential to address the limitations imposed by processing and post‐treatment through initial structural design. A critical open question is whether microstructural design can stabilize work‐hardening capability and alleviate premature local strain localization while remaining compatible with toughening mechanisms.

Inspired by natural selection and guided by biological structures, materials such as honeycomb structure MPEA,^[^ ^10^, ^11^ ^]^ nacre‐like structure titanium alloy,^[^ ^12^ ^]^ and mantis shrimp‐inspired magnesium alloy^[^ ^13^ ^]^ have been designed. Enamel, as a weapon for mice to quickly chew hard food, can resist a wide range of load stresses (≈0.45 to 2.5 GPa) and other shear stresses in the oral cavity.^[^ ^14^, ^15^ ^]^ Its alternating hard and soft nanosheet structure exhibits exceptional stress dissipation and crack resistance under complex loading conditions. This remarkable performance is attributed to its nanoscale structural parameters and the interface‐mediated rapid stress adaptation mechanism. Such interface‐mediated behavior alleviates stress/strain concentrations within the microstructure, enhancing both damage tolerance and strength, thereby offering critical insights for the design of biomimetic alloys. During the period under review in this work, the design of a material that mimics tooth enamel was reported by Lu et al.^[^ ^16^ ^]^

**Figure 1:**
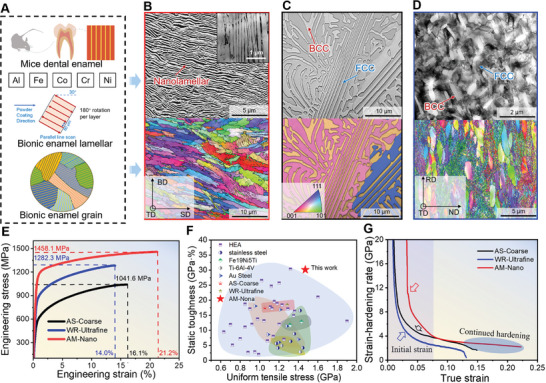
Enamel‐like microstructure and tensile mechanical properties of Co_17_Cr_16_Fe_16_Ni_34_Al_17_ multi‐principal elements alloys. A) Strategy for preparing MPEA using the L‐PBF method. B–D) The microstructure of the alloy was observed by overlaying Image Quality (IQ) and Inverse Pole Figure (IPF) maps obtained from EBSD. B) AM‐Nano exhibits a nano‐lamellar structure resembling enamel. C) AS‐Coarse shows a coarse‐grained state induced by casting. D) WR‐Ultrafine presents an ultrafine‐grained structure. E) Engineering stress‐strain curves at room temperature. F) Ashby plot of ultimate tensile strength versus static toughness. The current tensile performance of the AM‐Nano alloy stands out not only in comparison with similar severely plastically deformed metals but also with other high‐entropy alloys, titanium alloys, stainless steels, Fe‐Ni‐Ti alloys, and advanced high‐strength steels. G) Strain‐hardening rate versus true‐strain curves.

We have prepared a dual‐phase microstructure using multi‐principal element alloy (MPEA), which has parallel nano‐phase boundaries similar to those of mice enamel and is called AM‐Nano (**Figure** **1**A,B). Different from other nanostructured alloys, AM‐Nano can provide a window for stress threshold release in nanospace. It not only realizes continuous dislocation diffusion and stress transfer but also retains the toughening mechanism of dissipating crack tip energy, demonstrating stable strain‐hardening ability and mechanical functions of avoiding premature strain concentration. This alloy can obtain excellent tensile ductility of ≈21.2% under a high flow stress of ≈1458.1 MPa without cumbersome post‐treatments (such as heat treatment, high temperature, and high pressure, or hot isostatic pressing). There is no need for complex post‐processing, which reduces costs and processing complexity. Especially in the field of additive manufacturing, this directly formed high‐performance material has obvious industrial advantages. We prepare coarse‐grained (AS‐Coarse) and ultrafine‐grained (WR‐Ultrafine) samples by casting (Figure 1C) and warm rolling‐annealing (Figure 1D) to discuss the mechanical behavior and deep mechanism of AM‐Nano in comparison.

Simultaneously, we combined macro/micro in situ mechanical characterization with molecular dynamics (MD) simulations and density functional theory (DFT) calculations to capture the mechanical response of nano enamel MPEAs and reveal their microstructural mechanical behavior under external loads at scales from a few nanometers to hundreds of micrometers. We analyzed the evolution of dislocations and mechanical/functional properties. The source of the high strength and ductility/static‐toughness combination was fully demonstrated. Unlike nanocrystalline alloys that rely on small‐volume intragranular deformation, in enamel‐structured alloys, stable parallel phase boundaries activate atomic plane migration to form a mediated strengthening mechanism, promote the continuous activation of dislocations, provide a large amount of displacement plasticity space, and solve the contradiction that the nanocrystalline toughening mechanisms of deflecting, suppressing, and bridging microcracks are difficult to retain simultaneously, thereby widely alleviating strain localization and stabilizing the work‐hardening rate.

## 2 Results and Discussion

### 2.1 Uniting Tensile Ductility with Strength via Enamel‐Like Nanostructure

We first addressed two issues: one is the phase stability affected by fundamental physical parameters, such as mixing enthalpy,^[^ ^17^ ^]^ configurational entropy,^[^ ^18^ ^]^ atomic radius difference,^[^ ^19^ ^]^ and Gibbs free energy^[^ ^20^, ^21^ ^]^ (Table S1, Supporting Information). The second is the competitive growth of multi‐principal element phases influenced by undercooling during solidification. In Supplementary Texts 1–4, we calculated the possible combinations of Co*_x_* Cr*_x_* Fe*_x_* NiAl_1_ *_‐x_* + CoNiAl (*x* represents atomic concentration) based on solidification thermodynamics theory, concluding that the Co_17_Cr_16_Fe_16_Ni_34_Al_17_ system has low negative mixing enthalpy and significant comprehensive thermodynamic indices. These satisfy the conditions for phase separation and alternating distribution in solidification kinetics. Even under the high cooling rates of the AM process (Figure 1A), the phase structure of AM‐Nano remains stable. Using X‐ray diffraction (XRD) and electron backscatter diffraction (Figure 1B; Figures S1–S7, Supporting Information), we characterized the microstructure of the bulk AM‐Nano prepared by AM, validating our hypothesis. The material consists of a nano‐lamellar structure with face‐centered cubic (FCC) and body‐centered cubic (BCC) dual‐phase microstructures, featuring nearly parallel phase boundaries (Figure 1B), reminiscent of the strong and sharp structure of mice enamel (Figure S4, Supporting Information). Transmission electron microscopy (TEM) showed that the average thickness of the FCC phase is ≈140 nm, while the BCC phase is ≈70 nm, consistent with the Jackson‐Hunt rule^[^ ^22^ ^]^ described (Supplementary Text 4). These alternating phases form oriented, nearly equiaxed eutectic colonies, each with random crystal orientations (Figure 1B).

We report the macroscopic tensile results and the ductility/static‐toughness versus ultimate tensile strength Ashby relationship in Figure 1E,F (calculation method in Supplementary Text 5, 6). The enamel‐like nanostructured sample exhibits ultra‐high uniform tensile ductility of ≈21.2% and an unusual energy absorption capacity of ≈30.91 GPa·% static‐toughness under a high flow stress of ≈1458.1 MPa. It combines ductility comparable to that of AS‐Coarse with greater resistance to deformation failure than WR‐Ultrafine (Figure S5, Supporting Information). Interestingly, its energy absorption capacity increased by ≈72.2% to ≈84.3%. As shown in Figure 1F, our AM‐Nano alloy stands out from most previously reported MPEAs in terms of the balance between ultimate tensile strength and static toughness. This is a surprising result for a nano‐structured metal, avoiding the usual trade‐offs. This combination of strength and static toughness rivals that of third‐generation advanced automotive steels (≥20 GPa·%).^[^ ^23^, ^24^ ^]^ However, the latter requires complex processing techniques and post‐processing to obtain multiple phases or metastable structures, which are prone to Lüders band failure and ductile‐to‐brittle transitions.^[^ ^23^, ^24^ ^]^ Even compared to other various MPEAs,^[^ ^25^, ^26^, ^27^, ^28^, ^29^, ^30^, ^31^, ^32^, ^33^, ^34^, ^35^, ^36^, ^37^ ^]^ additive manufacturing eutectic alloys,^[^ ^38^ ^]^ steels,^[^ ^39^, ^40^, ^41^, ^42^ ^]^ and Ti alloys^[^ ^43^ ^]^ in the literature, AM‐Nano shows significant advantages.

Figure 1G presents the calculated strain‐hardening rate curves for these alloys. The significant differences between the strain‐hardening rate curves indicate the different responses of these alloys to external loads. WR‐Ultrafine and AS‐Coarse exhibit a sharp decline in strain‐hardening rate at the early stages of plastic deformation (indicated by arrows in Figure 1G) and premature plastic instability. In contrast, AM‐Nano does not become unstable due to the nanoscale crystal structure in the early and late stages of tensile plastic strain and exhibits stable strain‐hardening capability, prolonging uniform plastic deformation, and delaying the onset of necking instability.

### 2.2 Mitigating and Releasing Strain Concentration

**Figure 2:**
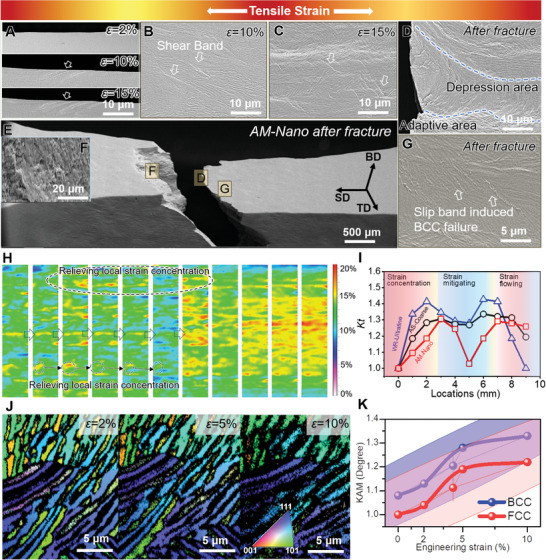
AM‐Nano's function of mitigating strain localization and the microfracture process. A–G) In situ SEM observation of deformation bands nucleation, propagation, and the mechanical response of the microstructure. H) Real‐time capture of the macroscopic strain evolution process using a high‐speed camera and digital image correlation (DIC) technology, revealing the sample's ability to mitigate local strain concentration. I) Extraction of local strain concentration from H) and its representation through changes in the equivalent circular strain concentration factor *K_t_*. J) In situ identification of phase changes under load using EBSD. K) Evolution of kernel average misorientation (KAM) during in situ EBSD process.

**Figures** **2**(A–G) and S8 (Supporting Information) show in situ tensile testing under SEM to examine the micro‐scale strain process of AM‐Nano samples to explore its microscale mechanical failure resistance response. Figure 2A indicates that severe deformation bands preferentially initiate at the free surface at 10% strain, similar to the crack formation behavior in other alloys.^[^ ^44^, ^45^, ^46^ ^]^ At this point, the lamellar structure oriented at ≈45° to the stress is favorable for shear band nucleation, while lamellae in other directions somewhat inhibit early strain progression. As strain continues to develop, the free surface deformation band propagates at 15% strain (Figure 2A,B). Shear bands within the microstructure begin to proliferate significantly across the constraints of other directional lamellar structures (Figure 2C), increasing the likelihood of micro‐crack nucleation within the microstructure. During the instantaneous fracture at the load limit of AM‐Nano (Figure 2D), the microstructure is twisted by stress, forming strain‐adapted zones on both sides and a central region of plastic flow‐induced depression, which helps delay the onset of plastic instability, enhancing the uniform elongation and adaptability to strain. The presence of high‐density dimples, steps, and intergranular fracture morphologies on the fracture surface of AM‐Nano (Figure 2F; Figure S9, Supporting Information) confirms that the sample underwent quasi‐cleavage fracture. Notably, near the fracture direction (Figure 2G), the BCC phase is sheared along the shear band direction and fails preferentially. Rather than the phase boundary cracking as commonly believed.

AM‐Nano's in situ SEM experiments revealed the destruction, twisting, and plastic flow phenomena of lamellar structures caused by shear strain under external loads, which provided a prerequisite for further revealing the mechanism of phase boundary‐mediated mechanism. The adaptability of stress and strain significantly influences the work hardening of these alloys. The evolution of macroscopic strain fields parallel to the external load direction was captured in real‐time using a high‐speed camera and digital image correlation (DIC) technology (Figure 2H; Figures S10–S12, Supporting Information), and the local strain concentration factor (*K_t_*) was extracted using the methods outlined in Supplementary Text 4, as shown in Figure 2I and Table S3 (Supporting Information). Interestingly, these samples all exhibited a trend of gradually decreasing local strain concentration to varying degrees (Figure 2H; Figure S10, Supporting Information), especially noticeable in AM‐Nano's significant alleviation of macroscopic strain localization in the early stages (Figure 2I), which may be related to the dual‐phase heterogeneity of the microstructure, a significant departure from other homogeneous materials.^[^ ^47^ ^]^

**Figure 3:**
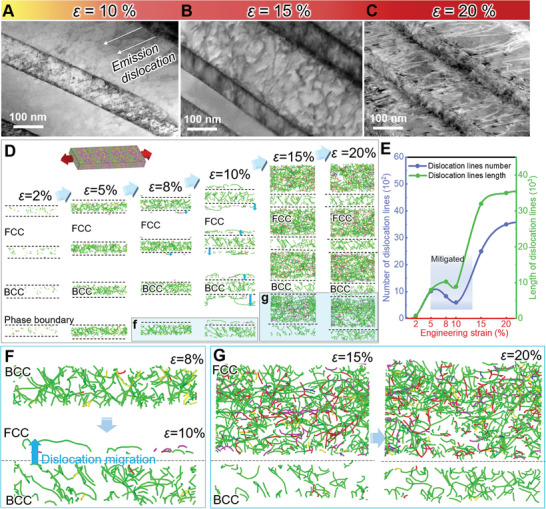
Semi‐in situ TEM and large‐scale molecular dynamics simulation results of AM‐Nano under uniaxial tensile deformation at different strains. A–C) Observation of microstructural changes in AM‐Nano at 10%, 15%, and 20% strains, respectively, during tensile deformation. D) The tensile process of AM‐Nano was simulated using large‐scale molecular dynamics to calculate the evolution of dislocations under quasi‐static tensile loading, showing high agreement with experimental results. From 5–10% strain, dislocations nucleate and proliferate in the FCC phase, while the accumulation of dislocations in the BCC phase decreases, indicating strain transfer between phases. The blue arrows of different lengths indicate the transfer of dislocations. The color of the lines represents different dislocation types. E) Dislocation line changes with increasing strain were extracted through MD simulations, showing an increase‐decrease‐reincrease trend. The dislocation lines start to saturate at 15% strain, leading to a decrease in the slope of the curve. F, G) Local magnification details of the individual layer structures of AM‐Nano at 8%, 10%, 15%, and 20% strains were examined. These images revealed the sequential nucleation of point defect dislocations on both sides of the interface and the transfer of dislocations from the BCC to FCC phases, collectively causing relief of dislocations in the BCC phase. These details help further clarify the nucleation and multiplication processes of dislocations in the BCC and FCC phases.

In situ, EBSD observations indicate that at 2–5% strain, the FCC phase exhibits minimal changes (Figure 2J; Figure S11, Supporting Information), with no significant substructure development, and stress is primarily concentrated in the BCC phase. Beyond 10% strain, both the FCC and BCC phases begin to exhibit simultaneous distortions. Kernel average misorientation (KAM) can qualitatively reflect the uniformity of microscale plastic deformation in materials.^[^ ^48^ ^]^ In the early stages of strain, the KAM values of the FCC phase in AM‐Nano are significantly lower than those of the BCC phase (Figure 2K), but they begin to shift to the FCC phase when the strain reaches 10%. The unique ability to mitigate strain localization and the mechanism of microscale strain distribution may contribute to the combination of high strength and ductility in AM‐Nano. Semi‐in situ TEM reveals that at 10% strain, the enamel‐like structure contains a large number of dislocations, which can accumulate and extend along the lamellar structure even within the limited nanoscale space (**Figure** **3**A–C). At this stage, dislocations begin to spread in the FCC phase. With increasing external load, dislocations in the BCC phase show little change, while the FCC phase undergoes major strain, entering a rapid dislocation accumulation phase (Figure 3B), consistent with in situ EBSD observations (Figure 2J; Figure S11, Supporting Information). At 20% strain, the dislocation densities in both phases reach saturation, and the BCC phase experiences severe deformation and plastic instability under shear stress (Figure 3C and Figure 2G).

We performed MD simulations to elucidate why dislocations can transfer between the two phases and remain significantly retained within the nanograins. Semi‐in situ TEM observations show that dislocations begin to proliferate in the FCC phase at 10% strain and approach saturation in subsequent stages (Figure 3A–C), which is highly consistent with the MD results (Figure 3D). This consistency helps clarify the microscale strain transfer and dislocation proliferation processes in AM‐Nano.

Due to the differing mechanical properties of the BCC and FCC phases, external loads initially promote dislocation accumulation within the BCC phase (at 2% strain). At 5–8% strain, the dislocation lines in the BCC phase significantly increase and become entangled. At this point, the BCC phase can no longer store additional dislocations, making the constraint layer slip mechanism difficult. The FCC phase then begins to activate dislocations (at 5–8% strain), transferring stress and strain to the FCC side. From the dislocation content (Figure 3D) and KAM values (Figure 2K), it is evident that the transfer effect is not yet significant at this stage, making it difficult to detect in experiments. The dynamic competition between the increase in FCC dislocation nucleation sites and the entanglement and annihilation of BCC dislocations leads to a reduction in the average number and length of dislocation lines in the system (Figure 3E).^[^ ^49^ ^]^ To accommodate deformation, dislocation lines suddenly cross phase boundaries into the FCC phase or undergo significant annihilation at 10% strain (Figure 3F), reducing the dislocation density in the BCC phase and significantly decreasing the number of dislocation lines in the system, providing ample slip space for subsequent strain (Figure 3G). This is called the phase boundary‐mediated mechanism. Therefore, this early strain localization mitigation mechanism and early strain‐hardening stability ability may benefit from the continuous dislocation nucleation and activation transfer under the mediated mechanism of BCC and FCC phase interfaces, thereby reducing dislocation accumulation.

### 2.3 Phase Boundaries‐Mediated Enhances Work‐Hardening and Crack Arrest Capabilities

**Figure 4:**
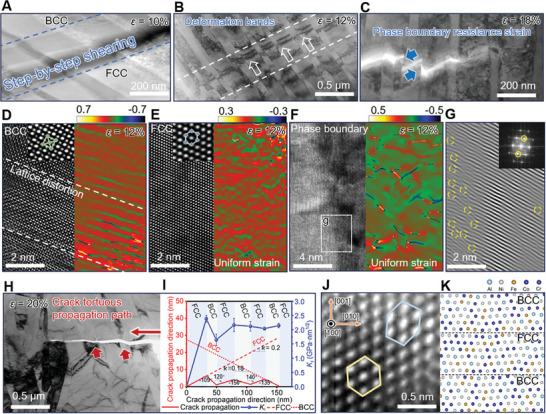
The mechanical response and failure modes of AM‐Nano during tensile deformation, characterized using semi‐in situ TEM. A–C) Microstructural interaction and microcrack formation states at 10%, 12%, and 18% strain, respectively. In (A) and (B), shear bands and slip traces left by shear stress in the matrix and phase boundaries are observed. (C) Shows microcracks emerging at 18% strain. High‐resolution transmission electron microscopy (HRTEM) reveals the lattice strain in the BCC matrix D), FCC matrix E), and at the phase boundaries F). Panel g identifies dislocation nucleation states on both sides of the phase boundary via local fast Fourier transform (FFT). At 20% strain, panel H) captures significantly extended zigzag cracks with inflection points at the phase boundaries. The local strain concentration factor distribution at the crack tip, calculated from the typical crack observed in panel G), is shown in panel I). Detailed characterization of the phase boundaries in J) facilitated the construction of the DFT model depicted in K).

To gain a deeper understanding of the toughening process mediated by phase boundaries, we used semi‐in situ TEM to characterize the crack propagation path and the microstructure of the surrounding deformation at different strain levels (**Figure** **4**). This allowed us to explore the “microstructure‐performance‐mechanism” relationship. High‐density phase boundaries separate the two phases with different mechanical properties. In the later stages of strain, external loads induce the formation of ≈200 nm wide shear deformation bands (Figure 4A), which easily leave deformation traces on the softer FCC phase (Figure 4B). Each time a shear band crosses a phase boundary, it deflects and propagates at a ≈45° angle to the loading direction. As shown in Figure 3C and S13, even when microcracks form through the shearing of phase structures, the interface forms a continuous bridge across the crack at the crack‐tip wake, preventing the instantaneous coalescence of cracks and thus exhibiting a crack‐tip bridging mechanism that resists crack opening.^[^ ^50^ ^]^ At a high strain of 18%, shear microcracks induce strong lattice strain concentration in the BCC matrix (Figure 4D), while the phase boundary accommodates the strain concentration caused by the crack, and the strain concentration begins to accumulate in the FCC (Figure 4E,F), and the nucleation of dislocations is observed (Figure 4G), which is consistent with the results of Figure 3D. Generally, BCC has fewer slip systems and anisotropic elastic modulus, which makes dislocation nucleation easier, while FCC makes it easier to generate stacking faults. At this strain, the dislocations in FCC may be transferred or activated to the FCC matrix on the other side due to the lattice strain of dislocations across the phase boundary in BCC (Figure 4F and Figure 4G). This may be due to the semi‐coherent relationship and small mismatch (only ≈2%) between the FCC and BCC phases (Supplementary Text 1). These nano‐scale phase boundary‐induced mechanisms ensure that before a coarse crack‐induced fracture occurs, the crack follows a nano‐scale tortuous propagation path (Figure 4H; Figure S13, Supporting Information), repeatedly dissipating the driving force of crack‐tip propagation and significantly suppressing crack strain concentration. The crack deflection and bridging mechanisms have been extensively studied in biological enamel,^[^ ^51^, ^52^ ^]^ indicating that the microstructure of AM‐Nano aligns well with biomimetic toughening mechanisms. According to linear elastic fracture theory Equation (1),^[^ ^53^ ^]^ the stress intensity factor (*K_I_*) at the crack tip is solely related to the type and shape of the crack.

(1) $$K_{I}=\sigma \sqrt{\pi \alpha }$$

where σ represents the nominal stress. Equation (1) gives the stress intensity factor of the straight center crack tip in the isotropic elastic infinite solids under plane strain conditions. According to the theoretical premises for an infinite space and elastic crack, 1) the crack in the sample is at the microscale, taking into account crack deflection at the crack tip, with a nanolayered structure in front. Assuming isotropic conditions, the crack would appear larger (as shown by the dashed line in Figure 4I). 2) Although there is an evident plastic zone in front of the crack tip, which is inconsistent with elastic conditions, this also suggests that if the crack were to propagate under elastic conditions, the resistance to its extension would be greater. The crack in Figure 4I fractures multiple phase domains under nominal stress of ≈1300, thus σ = 1300 MPa. *α* is half the length of the crack.

The crack in Figure 4H is quantified using Equation (1), yielding the data shown in Figure 4I. Influenced by the nanoscale phase boundaries, the crack path exhibits surprising tortuous turns with an average turning angle of ≈94.5°, enhancing the material's static fracture toughness. The small area under the curve and the reduced fluctuation range of the *K_I_* values indicate limited crack propagation, as the strain concentration at the crack tip is repeatedly restrained and unable to propagate effectively. The slope of the curve can predict crack propagation paths in equivalent single‐phase FCC (*k_FCC_* = 0.2) or BCC (*k_BCC_* = 0.18) structures, where cracks would be exceptionally large, highlighting the toughening advantage of the phase boundaries formed by the composite of solid solution and intermetallic compounds.

**Figure 5:**
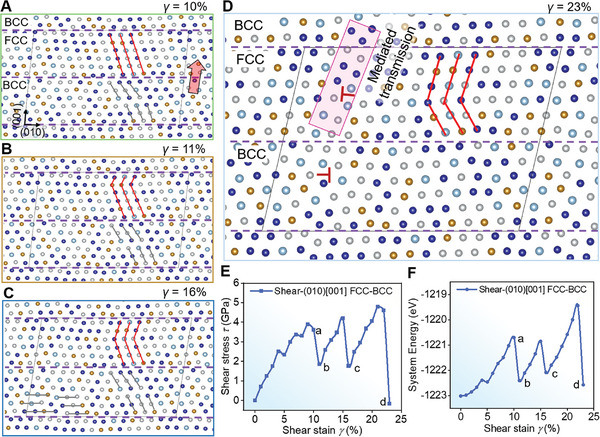
DFT stability evaluation of the AM‐Nano phase boundary. A–D) Structural evolution at characteristic positions during shear where energy fluctuations and stress release occur. A,B) At ≈10% to ≈11% shear strain, the first atomic layer near the phase boundary in the FCC phase is activated and slides perpendicular to the shear stress, causing significant energy dissipation and a sharp drop in shear stress. C,D) As shear strain increases, the second and third atomic layers in the FCC phase sequentially activate and slide, leading to continuous energy fluctuations that help alleviate internal stress and strain concentrations. At ≈16% strain C), dislocations begin to appear in the BCC phase. At ≈23% strain D), dislocation intensification is observed in the BCC phase, while dislocation accumulation starts in the FCC phase, with dislocations crossing the FCC/BCC phase boundary (indicated by the pink rectangle), transferring micro‐scale lattice strain. The three dashed lines represent the phase boundary formed by the dual‐phase structure stacking, with the parallelogram indicating the supercell used in DFT calculations. E) The relationship between shear stress and shear strain during shearing along (010)[001], with the evolution of the system's total energy shown in F). Arrows indicate the shear direction perpendicular to the phase boundary.

We conducted a series of DFT simulations to determine the mechanical origin of the phase boundary‐mediated toughening mechanism in AM‐Nano, which activates unique macro‐ and micro‐scale strain localization mitigation and crack‐arrest toughening mechanisms. As illustrated in Figure 4D–F, the lattice arrangement features of the BCC and FCC phases were examined. The expanded lattice models of BCC and FCC phases in space are shown in Supplementary Text 7 and Figure S13 (Supporting Information). Based on Figure 4, the local structures on both sides of the BCC/FCC phase boundary were constructed, as depicted in Figure 4K. To fully examine the lattice evolution mechanics of AM‐Nano, we performed calculations of pure FCC, pure BCC, and localized shear failure modes at the BCC/FCC phase boundary (**Figure** **5**A–D; Figures S14–S17, Supporting Information) based on the fracture shear failure parallel to and perpendicular to the phase boundary shown in situ SEM (Figure 2A–G) and semi‐in situ TEM (Figure 4).

Regardless of the presence of phase boundaries, HEAs of this composition began to significantly release shear stress (*τ*) at *γ* = ≈10–11% strain (Figure 5E; Figures S14–S17, Supporting Information), leading to the system's total energy stabilizing (Figure 5F). This phenomenon is fundamentally due to the statistical fluctuations in the composition and stacking arrangements of different atomic elements within the HEA lattice. In simulations containing phase boundaries, significant energy release to counteract shear stress continued to post ≈11% strain. As depicted in Figure 5A–D, the combined matrix can sequentially activate local atomic planes in the FCC phase, while the BCC phase sees the synergistic nucleation and proliferation of nanoscale dislocations. Due to the slight mismatch at the phase boundaries, sliding is not preferentially initiated. At higher strain levels, *γ* = ≈23%, to sustain energy dissipation and maintain structural stability, dislocations will traverse the phase boundary. These steps are the fundamental source for the phase boundary to exert a mediated mechanism, enabling AM‐Nano to have the main mechanisms of mitigating strain concentration and dissipating the vertical shear energy of cracks. This observation aligns with the captured dislocation crossover process in samples at ≈15% strain. This leads to non‐localized plastic strain spreading across a wide spatial range, achieving exceptional ductility and static toughness at high strength. We also conducted simulations by altering the direction of the applied force to explore the directional dependence of interfacial shear sliding and its impact on interfacial stability and system energy absorption efficiency. We found that the shear stress direction did not fundamentally alter the plastic mechanisms and mediated process activation of the matrix lattice and phase boundary (Figure S17, Supporting Information).

## 3 Conclusion

In summary, we have broken the traditional contradiction between alloy strain concentration, plastic instability, and tensile strength‐ductility toughening adjusted through processes and post‐treatments. We achieve bionic microstructure design in a single manufacturing process and realize the combination of strength and static toughness. At a high flow stress of ≈1458.1 MPa, its static toughness is ≈30.91 GPa. Reduce processing costs caused by post‐treatment. Through the comparison and combination of in situ experiments and theoretical calculations, it is revealed that the stable phase interface in bionic alloys is conducive to the mediated strengthening mechanism caused by atomic‐activated migration. On the one hand, the mediated strengthening mechanism provides a large amount of displacement plasticity space for the nucleation, expansion, and coalescence of dislocations, promotes the transfer of dislocations to the other phase on both sides through short‐spacing phase boundaries for continuous activation, reduces the accumulation of lattice defects and strain concentration, thereby promoting continuous work‐hardening ability. This provides early relief for the local strain localization of alloys. On the other hand, the mediated strengthening mechanism continuously activates the deflection, suppression, and bridging mechanisms of microcracks, enabling the strain threshold to be transmitted along the phase boundary and even penetrate the other side. It synergistically activates a series of displacement deformations on both sides of the phase boundary, provides more energy dissipation channels, and effectively alleviates the strain localization caused by crack tips, showing excellent energy absorption capacity. These findings provide a promising example for customizing the macroscopic mechanical response and physical properties of nanostructures and enhance our fundamental understanding of the potential mechanical behaviors of nanostructured materials.

## 4 Experimental Section

### Materials

The AM‐Nano samples with a chemical composition of Co_17_Cr_16_Fe_16_Ni_34_Al_17_ were manufactured using a BLK series L‐PBF machine, custom‐made by Foshan Chenfeng Material Technology Co., Ltd., and Bright Laser Technologies. This machine is equipped with a Yb fiber laser with a maximum power of 500 W. The powders were prepared by Foshan Chenfeng Material Technology Co., Ltd. using gas atomization, with particle sizes ranging from 15 to 53 µm (average size 30 µm), and were used to construct alloy plates with dimensions of 40 mm (length) × 10 mm (width) × 2.1 mm (thickness). During bi‐directional printing, 4140 steel was used as the substrate, with a scanning speed of 1000 mm s^−1^, a layer thickness of 40 µm, scan spacing of 80 µm, and rotation angles as shown in Figure 1A.

The AS‐Coarse samples of the same composition were cast in a vacuum induction furnace, undergoing five uniform melting cycles. Alloy plates with dimensions of 40 mm (length) × 10 mm (width) × 3.1 mm (thickness) were cut from the AS‐Coarse samples using electrical discharge machining and then homogenized at 1200 °C for 3 h. The samples were heated to 800 °C in a box‐type heat treatment furnace and held for 20 min to ensure uniform temperature distribution. Immediately after, they underwent warm rolling plastic deformation with an 80% reduction rate and were water quenched, resulting in a final thickness of 2.48 mm. Finally, the samples were annealed at 800 °C for 1 h to produce the WR‐Ultrafine samples.

### Characterization

In situ tensile tests are completed in the TESCAN S8000 ultra‐high resolution field emission scanning electron microscope (SEM). Metal plates are processed into dumbbell‐shaped specimens of 2 mm (length) × 1.5 mm (width) × 1 mm (thickness) for in situ scanning electron microscope (SEM) and in situ electron backscatter diffraction (EBSD) tensile tests. Before applying load, the surface of the sample is subjected to stress relief treatment by an argon ion polishing device developed by the Institute of Geology and Geophysics, Chinese Academy of Sciences. During the loading process, the sample is stretched at a strain rate of 5 × 10^−4^ s^−1^ under the control of a high‐precision lead screw and transmission device. Through servo and load control, the microstructure morphology data is collected every time the strain of the sample increases by 2% until the specimen necks down and fails.

Microstructural and defect evolution was characterized using a transmission electron microscope (Talos F200X system) operating at 200 kV. The AS‐Coarse sample exhibits coarser FCC and BCC phases (Figure 1C), not only showing alternating lamellar structures but also large irregular dendritic features. The average sizes of the FCC and BCC phases are ≈6.89 and 3.97 µm, respectively. The AS‐Coarse sample was warm‐rolled at 800 °C, reducing the sample thickness by 80%, followed by annealing at 800 °C for 1 h. Under the combined effects of temperature and high rolling forces, the BCC phase fractured along its length into short rod‐like structures, uniformly dispersed within the FCC matrix (Figure 1D). The ultrafine BCC phase along the length direction has a size of ≈0.97 µm. The phases in all three samples exhibited heterogeneous structural characteristics. The dual‐phase structure of the bulk alloy was experimentally identified using X‐ray radiation, with a scanning range of 20–100° and a step size of 2° min^−1^.

### Testing

The wire‐cutting equipment is used to cut the samples with three‐grain sizes into dumbbell‐shaped tensile specimens of 10 mm (length) × 2 mm (width) × 1 mm (thickness). The specimens are placed on the MTS universal mechanical testing machine equipped with digital image correlation (DIC) technology to conduct quasi‐static room‐temperature uniaxial tensile loading tests at a strain rate of 5 × 10^−4^.

Before mechanical loading, a uniform white paint base is sprayed on the surface of the sample, and the deformation information is marked by using a black paint speckle pattern. The process of applying load is captured by a high‐speed camera at a frame rate of 500 ms to record the displacement of the speckles. The overall strain distribution of the sample is calculated by VIC‐2D and GOM software according to the photo data. The size of the selected calculation unit is 15 × 15 pixels.

The mechanical properties of AM‐Nano exhibit significant advantages. The cast state AS‐Coarse has a coarse grain size, resulting in lower yield strength (≈523.7 MPa) and ultimate tensile strength (≈1053.7 MPa), with a uniform elongation of ≈16.6%. When the microstructure is refined to the ultrafine grain level, even after a short heat treatment of 1 h, a high density of defects can still be retained, leading to strain localization and a strength‐ductility trade‐off. Although the yield strength increases to ≈937.5 MPa and the ultimate tensile strength rises to ≈1284.3 MPa, the ductility decreases by ≈14% compared to AS‐Coarse, resulting in a remaining uniform elongation of only ≈14.4%. The static toughness of WR‐Ultrafine and AS‐Coarse is ≈18.49 GPa·% and ≈17.49 GPa·%, respectively.

### Simulation

A series of simulations of uniaxial stretching of HEAs were performed using the large‐scale atomic/molecular massively parallel simulator (LAMMPS).^[^ ^54^ ^]^ A 4‐layer supercell consisting of FCC and BCC was constructed (500 000 atoms), deformed along the fcc (111) direction. They were first subjected to equilibrium by dynamic relaxation for 200 ps at 300 K and 0 bar using the NPT functional and conjugate gradient scheme, and then stretched to 20% strain at 300 K. Visualization was performed using OVITO.^[^ ^55^ ^]^ Dislocation analysis (DXA)^[^ ^56^ ^]^ was used to characterize the type and number of dislocations. The embedded atom method (EAM) developed by Farkas and Caro^[^ ^57^ ^]^ was used to describe the interactions in the Fe‐Cr‐Co‐Al‐Ni HEA. This EAM has been successfully used to simulate the deformation behavior of Fe‐Cr‐Co‐Al‐Ni nanolaminates,^[^ ^58^ ^]^ and high entropy alloys.^[^ ^59^ ^]^ The simulation results are in agreement with the experimental results. The time step is fixed to 1 fs. During the tensile test, the temperature was kept constant at 300 K by the NPT ensemble. The atoms were colored by the centrosymmetry parameter method. The strain rate was 10^9^ s^−1^ and the total applied strain was 20%. Additive manufactured metals often bear high solidification‐induced internal stresses, potentially resulting in an initial dislocation density within the sample. This simulation aims to analyze the processes of dislocation multiplication and propagation, as well as the interactions between the matrix and phase boundaries. Therefore, the influence of initial dislocations is disregarded here.

The first‐principles density functional theory (DFT)^[^ ^60^ ^]^ calculations were carried out by using the Vienna ab initio simulation package (VASP) code,^[^ ^61^, ^62^, ^63^, ^64^ ^]^ The interaction between electrons and ions were treated by the projector augmented wave (PAW) and special quasi‐random structure (SQS) method.^[^ ^65^ ^]^ The exchange and correlation energies of electrons were described by PBE pseudopotentials^[^ ^66^ ^]^ within generalized gradient approximation (GGA).^[^ ^67^ ^]^ The electronic wave functions were expanded with a plane‐wave basis set up to a kinetic cutoff energy of 350 eV which is 1.3 times higher than the default value for all elements in the alloy. All the atoms were fully relaxed until the total energy and the Hellmann‐Feynman force on atoms were all less than 1 × 10^−4^ eV and 0.05 eV Å^−1^, respectively. An 11 × 1 × 1 *k*‐point grid was employed for the Brillouin zone sampling of the supercell including 186 atoms. To construct a periodic supercell containing an interface between FCC and BCC high entropy alloys CrNi_2_CoAlFe, a 96‐atom FCC‐like and a 90‐atom BCC‐like special quasirandom structure (SQS)^[^ ^68^ ^]^ supercells were constructed. The relaxed lattice constants were *a* = 2.55 Å, *b* = 34.21 Å, *c* = 11.84 Å and *a* = 2.52 Å, *b* = 34.53 Å, *c* = 12.30 Å for the fcc‐like and the BCC‐like supercells, respectively. The interface was then generated by stacking the FCC‐like and the bcc‐like supercells along the ***c*** direction alternatively, leading to a lattice mismatch of below 1% along *b*.

The uniaxial shear loading on the supercell is simulated here through a quasi‐static displacement‐controlled method,^[^ ^69^, ^70^ ^]^ where the lattice vectors of the supercells are deformed incrementally by shear strains in the *x*‐direction on the shear plane perpendicular to the *z*‐direction. A shear strain step of Δ*γ*_xz_ = 0.01 is adopted. At each strain step, the applied shear strain *γ*_xz_ is fixed to calculate the corresponding shear stress *τ*_xz_, and the other five independent elements of the strain tensors and the positions of all atoms inside the supercells are fully relaxed. The lattice constants of the sheared supercells, the atomic positions, and the shear stress‐strain relation (*τ*_xz_‐*γ*_xz_) are calculated at each step by this constrained relaxation.

## Conflict of Interest

The authors declare no conflict of interest.

## Supporting information



Supporting Information

## Data Availability

The data that support the findings of this study are available in the supplementary material of this article.
