# Supplementary material for: Mitigating Strain Localization via Stabilized Phase Boundaries for Strengthening Multi‐Principal Element Alloys
Source: Adv Sci (Weinh). 2025 Mar 8;12(17):2414783. doi: 10.1002/advs.202414783 (PMC12061292; doi:10.1002/advs.202414783)
Supplement: Supplementary file 1 — Supporting Information [file ADVS-12-2414783-s001.docx]

Supporting Information

Mitigating Strain Localization via Stabilized Phase Boundaries for Strengthening Multi-principal Element Alloys

*Jinliang Du†, Shukuan Guo†, Hangqi Feng, Weijie Li*, Zhixin Huang, Zhongji Sun, Yunli Feng, Pei Wang, Ying Li**

*Corresponding author. Email: wj.li@bjtu.edu.cn (W. L.); bitliying@bit.edu.cn (Y. L.)

†These authors contributed equally to this work.

Supplementary Text 1 Description of background and phase composition

Supplementary Text 2 Phase size calculation method

Supplementary Text 3 Phase stability evaluation method based on element parameters

Supplementary Text 4 Phase stability evaluation method based on crystal alternating growth dynamics

Supplementary Text 5 Strain distribution

Supplementary Text 6 Description of tensile static-toughness

Supplementary Text 7 DFT calculation of phase boundary shear resistance

Figs. S1 to S17

Tables S1 to S3

Supplementary Text 1 Description of background and phase composition

Multi-principal element alloys (MPEAs), due to their nearly limitless multi-component design space, exhibit astonishing mechanical properties ^[1]^. This provides hope for solving the strain concentration problem. The basic plastic deformation mechanism of the toughened MPEA reported so far is similar to that of conventional metals ^[2,3]^. Typically, there appears to be an inevitable trade-off between strength and ductility/toughness during grain refinement ^[3]^. This trade-off is not surprising and is closely related to strain concentration, as ultrafine/nanocrystalline (UFG/NC) structures produced from coarse-grained MPEAs and severe plastic deformation (SPD) often come with substructure formation, which easily initiates strain concentration at the same locations ^[4]^. Theories such as dynamic recovery ^[5]^, dislocation annihilation ^[6]^, plastic instability ^[7]^, and high initial dislocation density ^[8]^ suggest that UFG/NC MPEAs experience a sharp decline in stress they can withstand post-yield, leading to highly localized grain boundary shear fractures or brittle tearing. This undesirable brittleness or low ductility stems from the limited proliferation and interaction of dislocations within a small volume, inhibiting continuous nucleation of new dislocations and sustained activation of displacement deformation mechanisms, causing strain localization, plastic instability, and premature failure ^[9]^. Therefore, an open question is the lack of work hardening or premature local strain concentration in UFG/NC materials.

High temperature/pressure or heat treatment techniques improve the work-hardening performance of materials by causing grain boundaries and the interior of crystals to rearrange due to the temperature effect, thus altering the temperature and time to return the material to a uniform, softened state. Potential strain concentration sites within the material are also alleviated ^[10]^. However, the recovery and recrystallization (or growth) of grains generally reduce the strength or lead to the precipitation and growth of second-phase particles at grain boundaries and other high-energy locations, forming new local strain concentration sites ^[11]^. Therefore, balancing these issues to mitigate or avoid local strain concentration is challenging. Enhancing work-hardening ability and eliminating or mitigating strain concentration has become a long-term challenge in the design of strengthened and toughened structural materials.

In recent years, researchers have introduced gradient structures (GS) into single-phase coarse-grained metals from a structural design perspective. This is achieved through severe plastic deformation (SPD) methods such as cold rolling ^[12]^, warm rolling ^[13]^, torsion ^[12]^, and surface grinding ^[8]^, which induce continuous hardening behavior to transmit micro-scale strain. The preparation of UFG/NG/GS metals involves multiple steps, including casting, plastic deformation, heat treatment, and surface treatment. Additive manufacturing (AM) features high solidification and rapid forming characteristics ^[14]^. Biphasic materials have a high interface density and different mechanical properties in the two phases, allowing stress and energy transfer through the phase boundaries during interaction ^[15]^. These provide the possibility of achieving the same goals without complex plastic processing and post-processing, all within a single manufacturing process.

Therefore, it is necessary to design a mechanical mechanism that imparts stable strain-hardening capability to initial nanocrystals without post-processing, thereby avoiding plastic instability. We address this challenge by using AM technology (**Fig. 1A**) to prepare a dual-phase microstructured Co_17_Cr_16_Fe_16_Ni_34_Al_17_, MPEAs, referred to as AM-Nano, resembling mice dental enamel (**Fig. 1B**). This structure mitigates local strain concentration at macro, micro, and crack-tip scales. In the supplementary information, we mainly show the design idea of composite bionics of soft structure FCC and hard structure BCC, macro/microscale fracture modes, and atomic-level simulation data. First, based on systematic energy assessment and screening of element ratios, and then configuring the alloy system with the characteristics of individual elements. Then, based on the phase precipitation theory during alloy solidification, evaluate the stability of the alloy during the high cooling rate in the additive manufacturing process to ensure the alternating precipitation of FCC and BCC at room temperature. Finally, in situ experiments and simulation calculations, reveal the strain state of the material under fracture during the crack failure process.

We confirmed that the microstructure of AM-Nano consists of dual-phase FCC and BCC structures through the calibration of XRD diffraction peaks (**fig. S1**). The lattice constants $a$ of FCC and BCC are 3.590 and 2.871, and the lattice mismatch degree $\Delta a$ is only ~2%, where $\Delta a=2(a_{FCC}-a_{BCC}/cos\theta)/(a_{FCC}+a_{BCC}/cos\theta)$. Combined with EBSD analysis, it was found that these dual-phases form clusters with consistent orientation, resembling the structure within a single cluster of mouse enamel (**fig. S2**). Nature has evolved outstanding structural features in organisms through environmental selection, guiding the direction of materials design ^[16],^ ^[17],^ ^[18]^. Mouse teeth, protected by enamel, can gnaw on hard objects such as nuts and wood at high speeds, far surpassing human teeth. We aim to achieve competitive alternate precipitation of BCC and FCC phases through additive manufacturing deposition. According to the Jackson-Hunt theory ^[19]^ and the Hume-Rothery rules ^[20]^, this requires meeting the conditions of phase structure stability and supercooling. The Hume-Rothery rules identify atomic size difference (*δ_r_*) and mixing enthalpy (Δ*H_mix_*) as two key factors affecting phase stability. The Jackson-Hunt theory explains the issues of competitive alternate growth of microstructures in alloys. It links lamella spacing to growth dynamics at supercooling.

CoCrFeNi is the FCC-forming element and NiAl is the main BCC element. The overall content of CoCrFeNi is taken as *x* (*x*=0, 0.1, 0.2, …, 1) to screen the alloy composition. Make small adjustments and optimizations through single elements. **Figure S4A** shows that the solid phase $\Delta H_{mix}^{\emptyset}$ after combining NiAl-BCC with the classical CoCrFeNi-FCC is less than 10 kJ/mol. If this alloy meets the physical requirements of MPEA, there is no significant segregation or chemical bonding tendency between the atoms of its components, the contribution of each component to the mixing enthalpy is uniform, the interaction forces between different elements are similar, and the chemical potential in the system may be uniformly distributed. The mixing enthalpy of (CoCrFeNi)*_x_*(NiAl)_1_*_-x_* is small, which helps to form a dual-phase or multi-phase microstructure. According to the principle of symmetrical nearest neighbor interaction, this phenomenon implies that the mixing enthalpy changes little and there is no large-scale endothermic or exothermic effect.

Assuming that the interactions between all elements are equivalent, it can be seen from **fig. S4B** that the configurational mixing entropy ${\Delta S}_{mix}^{\emptyset}$ mainly depends on the concentration ratio $c_{i}$ of the different elements, and is independent of the specific types of elements, environmental temperature, and constituent classification. The ${\Delta S}_{mix}^{\emptyset}$ of the alloys increases first and then decreases as *x* increases. At *x*=0.4-0.9, ${\Delta S}_{mix}^{\emptyset}$ has a higher performance.

We found that the *VEC* of alloys ranges between 6 and 8.5, which meets the stability requirements for MPEAs. Notably, there is a significant fluctuation in *VEC* at *x*=0.1 and 0.8, which may cause changes in system stability. In alloys and complex metallic compounds, fluctuations in *VEC* indicate abrupt changes in electronic distribution and bonding characteristics between atoms, which can affect the crystal structure and phase stability of the materials.

The stability of the system is determined by the mixing of Gibbs free energy and the interaction parameters between the components. **Supplementary Figure S4** shows the free energy at the solid-liquid interface front after the two are combined, which helps us to preliminarily evaluate the spontaneity and stability of the system during solidification at the solid/liquid interface front. Based on the basic law of thermodynamics, Equation S2, the system tends to spontaneously transfer from a high free energy state to a low free energy state, and ΔG<0 is the prerequisite for spontaneous precipitation of the solid phase in the absence of other driving forces during aging and cooling. (CoCrFeNi)*_x_*(NiAl)_1_*_-x_* alloys have negative free energy at the solid-liquid interface front, and $G_{mix}^{\emptyset}<G_{mix}^{l}<0$. In a system, a lower Gibbs free energy means that the system is in a more stable state. The system always tends to reach the lowest free energy state, which represents the equilibrium and the most stable configuration of the system. The more negative the free energy, the more sufficient the driving force for solid-phase precipitation. As the content of CoCrFeNi increases, the driving force of (CoCrFeNi)*_x_*(NiAl)_1_*_-x_* decreases, and the driving force of (CoCrFeNi)*_x_*(NiAl)_1_*_-x_* increases.

In addition, An et al. ^[21]^ showed that the more negative $\Delta H_{mix}^{\emptyset}$ and the higher the physical parameters of ${\Delta S}_{mix}^{\emptyset}$, *VEC*, and *δ_r_*, the more conducive it is for the system to precipitate a stable solid phase. The product of ultimate tensile strength and uniform elongation (*PSE*) ^[22]^ is often used to evaluate the comprehensive properties of materials. To quickly screen alloys that meet the requirements of being stable at equilibrium position and room temperature, the key parameters ${\Delta S}_{mix}^{\emptyset}$, *VEC* and *δ_r_* are simply multiplied by the calculation method of *PSE* to obtain the comprehensive index *SVRE* (kJ·mol^-1^·K^-1^·%), as shown in Equation S16.

$SVRE={\Delta S}_{mix}^{\emptyset}\times{VEC\times\delta}_{r} (\Delta H_{mix}^{\emptyset}<0, \Delta G_{mix}^{\emptyset}<0)$ (S16)

(CoCrFeNi)*_x_*(NiAl)_1-_*_x_* satisfies the prerequisites of spontaneous transformation and negative $\Delta H_{mix}^{\emptyset}$, resulting in the relationship between *SVRE* and *x* as shown in **Supplementary Figure S4, S5**. In addition to the *x*=0.8 alloy that does not meet the VEC condition, there is also a (CoCrFeNi)_0.5_(NiAl)_0.5_ alloy with high SVRE of *x*=0.5 in the (CoCrFeNi)*_x_*(NiAl)_1-_*_x_* system. In the designed alloy, considering that the internal stress in the solidification of additive manufacturing is difficult to release, which may cause the instability of the flow of elements at the solid-liquid front, a small amount of Ni is added to the obtained proportional variant (CoCrFeNi)_16_(NiAl)_16_ to stabilize the FCC structure. To avoid destroying the above thermodynamic relationship, Al and Co, which have the smallest difference in atomic radius and valence electron concentration and a higher gap with Ni, are added to promote the stable formation of NiAl, taking into account the strength of the alloy, and enhance the stability of the BCC crystal structure. Therefore, the (CoCrFeNi)_16_(NiAl)_16_+Ni_2_CoAl alloy, namely Co_17_Cr_16_Fe_16_Ni_34_Al_17_, is obtained.

Supplementary Text 2 Phase size calculation method

The phase size identification was performed using the Grain Size (Diameter) module in the OIM software for EBSD analysis. This functionality merges pixels within the same phase into a single unit and assigns an independent label. The area (A) of each phase is calculated by summing the pixel points belonging to the grain. Assuming the phase is circular, the size is calculated using the formula:

$$D=\sqrt{\frac{4A}{\pi}}$$

By selecting the FCC or BCC phase, their respective sizes were calculated. We also conducted additional EBSD measurements at different locations to further verify the accuracy of the statistics. The FCC phase size was determined to be 6.89 ± 0.3 μm, while the BCC phase size was 3.97 ± 0.3 μm.

Supplementary Text 3 Phase stability evaluation method based on element parameters

This work proposes a nano-bionic design method that differs from traditional alloy design by no longer considering a single element as the endpoint of a binary phase diagram, but instead considering the constituent elements of BCC and FCC as a whole. Thus, if the entire system stably exists, it meets the conditions for forming an MPEA. When the liquid phase (*l*) is cooled to the critical precipitation temperature (*T_m_*), the liquid phase *l* will transition to two stable solid phases, composed of BCC and FCC, under equilibrium conditions. If one phase has a stronger ability to reduce the Gibbs free energy of the alloy system, it indicates that this phase is more likely to precipitate from the matrix during rapid solidification or aging, implying lower stability of the liquid phase *l* in the system. This study considers the dynamic equilibrium of the liquid phase *l* and solid phase $\emptyset$ at *T_m_* as the basic environment for evaluating the phase stability of MPEAs. If the Gibbs free energy of the two precipitated solid phases (${\Delta G}_{mix}^{\emptyset}$) is lower than that of the liquid phase (${\Delta G}_{mix}^{l}$), it indicates that the stability of the liquid phase *l* is lower, and the system can spontaneously transform into the BCC and FCC phases, stably existing at room temperature through the solidification process. Conversely, if the precipitation conditions are not met, the system tends to exist in a single-phase or unstable form. The Gibbs free energy difference ${\Delta G}_{mix}={\Delta G}_{mix}^{l}-{\Delta G}_{mix}^{\emptyset}$ between the frontier liquid phase *l* and solid phase $\emptyset$ in the system, can be expressed by Eq. S1, S2.

${\Delta G}_{mix}^{l}=\sum_{i=1}^{n} c_{i}G_{i}^{Pure}+RT_{m}\sum_{i=1}^{n} c_{i}lnc_{i}$ (S1)

${\Delta G}_{mix}^{\emptyset}=\Delta H_{mix}^{\emptyset}-T_{m}{\Delta S}_{mix}^{\emptyset}$ (S2)

Where $c_{i}$ is the atomic concentration of element *i*. $G_{i}^{Pure}$ is the Gibbs free energy of pure element *i* in the liquid state. *R* is the gas constant. $\Delta H_{mix}^{\emptyset}$ and ${\Delta S}_{mix}^{\emptyset}$ represent the mixing enthalpy and configurational mixing entropy of the two solid phases $\emptyset$, respectively. An et al. ^[23]^ pointed out that mixing enthalpy can decrease system stability by promoting the precipitation of intermetallic compounds. They argued that a negative mixing enthalpy favors the formation of intermetallic compounds and hinders the formation of a complete solid solution phase. This indicates that the stronger the bonding force between elements, the more the Gibbs free energy tends to decrease, thus promoting the formation of intermetallic compounds.

In the conventional thermodynamic model, the mixing enthalpy $H_{mix}^{\emptyset}$ of MPEAs is defined in Eq. S3. The Miedema model ^[24]^ suggests that the interaction parameter $\Omega_{ij}^{\emptyset}$ has a relationship with the mixing enthalpy $\Delta H_{mix}^{\emptyset}$ between equimolar elements, as shown in Equation S4. $\Delta H_{mix}^{\emptyset}$ is mainly generated by the interaction between elements, and for the same element, $\Delta H_{mix}^{\emptyset}$=0. For binary systems, if there is an attractive force between two elements, $\Delta H_{mix}^{\emptyset}$ is negative; if there is a repulsive force, it is positive. Zhang et al. ^[25]^ reviewed the rules for MPEA formation and found that the range for $\emptyset$ formation is broad, with $\Delta H_{mix}^{\emptyset}$  < 10 kJ/mol. The bond enthalpy ($\varepsilon_{ij}^{\emptyset}$ and $\varepsilon_{ii}^{\emptyset}$) between same or different atom pairs is estimated from first principles calculations, as shown in Eqs. S5 and S6. The mixing enthalpy information between equimolar elements is shown in **Tables S1 and S2**.

$\Delta H_{mix}^{\emptyset}=\sum_{i=1}^{n} \sum_{j=i+1}^{n} \Omega_{ij}^{\emptyset}x_{i}x_{j}$ (S3)

$\Omega_{ij}^{\emptyset}=4\Delta H_{ij}^{\emptyset}$ (S4)

$\varepsilon_{ii}^{\emptyset}=-\Delta H_{v}^{\emptyset}/D_{ii}^{\emptyset}$ (S5)

$\varepsilon_{ij}^{\emptyset}=\left( \frac{1}{D_{ij}^{\emptyset}} \right)\left( -\Delta H_{v}^{\emptyset}-\varepsilon_{ii}^{\emptyset}D_{ii}^{\emptyset}-\varepsilon_{jj}^{\emptyset}D_{jj}^{\emptyset} \right)$ (S6)

where $D_{ii}^{\emptyset}=NZ_{ii}^{\emptyset}/2$ represents the number of bonds per mole of pure metal, $Z_{ii}^{\emptyset}$ represents the coordination number of pure metal, *N* is Avogadro's constant, and $D_{ii}^{\emptyset}$, $D_{jj}^{\emptyset}$ and $D_{ij}^{\emptyset}$ represent the number of *ii* bonds, *jj* bonds and *ij* bonds per mole of alloy, respectively. $\Delta H_{v}^{\emptyset}$ refers to the enthalpy difference of one mole of condensed alloy.

For a random system containing *N* (i=1, 2, 3, …, *n*) components, its configuration mixing entropy ${\Delta S}_{mix}^{\emptyset}$ is expressed as Eq. S10.

${\Delta S}_{mix}^{\emptyset}=-R\sum_{i=1}^{n} c_{i}lnc_{i}$ (S10)

According to the Hume-Ruthery rule ^[20]^, in addition to $\Delta H_{mix}^{\emptyset}$ and ${\Delta S}_{mix}^{\emptyset}$, the valence electron count *VEC* and the atomic size difference *δ_r_* are also the main factors affecting the stability of the system. Chanda et al. ^[26]^ pointed out that when 6≤*VEC* and *δ_r_* > 3%, the dual phase tends to be stable. These parameters are defined in Eqs. S11 and S12.

$\delta_{r}=\sqrt{\sum_{i=1}^{n} c_{i}{(1-r_{i}/(\sum_{i=1}^{n} c_{i}r_{i}))}^{2}}$ (11)

$VEC=\sum_{i=1}^{n} c_{i}{VEC}_{i}$ (12)

where *r_i_* is the atomic radius of the *i*th component and *VEC_i_* is the valence electron count of the individual element.

Supplementary Text 4 Phase stability evaluation method based on crystal alternating growth dynamics

Jackson and Hunt ^[27]^ observed that the eutectic spacing (*λ*) corresponding to the minimum undercooling follows the relationship in Eq. S17. Hillert ^[24]^ noted that the Jackson-Hunt rule is also applicable to alloys with lamellar microstructures.

$\lambda^{2}v=\frac{K_{2}}{K_{1}}$ (S17)

where *v* represents the growth rate., while *K_1_* and *K_2_* are constants at the liquid-solid interface during the solidification process.

The relationship expression between the isothermal interface average undercooling, growth rate, and eutectic spacing is:

$\Delta T=K_{1}v\lambda+\frac{K_{2}}{\lambda}$ (S19)

Given the minimum undercooling $\Delta T$, the two equations can be combined to obtain the lamellar thickness that satisfies the condition for the lamellar alternating growth microstructure. Therefore, the alloy we designed satisfies the undercooling and thermodynamic conditions at the liquid-solid interface, allowing FCC and BCC phases to alternate in a manner similar to the structure found in mouse tooth enamel.

According to Senninger and Voorhees ^[28]^:

$K_{1}=(\frac{-m_{2}^{FCC}m_{2}^{BCC}}{\Delta m_{2}f_{FCC}f_{BCC}})Q\frac{\Delta C_{2}}{D_{2}}$ (S20)

$K_{2}=\frac{-2m_{2}^{FCC}\Gamma_{BCC/i}sin(|\theta_{BCC}|)}{f_{BCC}\Delta m_{2}}+\frac{2m_{2}^{BCC}\Gamma_{{FCC}/i}sin(|\theta_{FCC}|)}{f_{FCC}\Delta m_{2}}$ (S21)

where *m* is the slope of the liquidus surface. *Q* is a constant, *ΔC* is the change in alloy element concentration. *f* is the volume fraction of each phase. *D* is a constant, the diagonal term of the interdiffusion coefficient matrix of element *i*. *θ* is the angle between the solid phase and the liquid phase during solidification precipitation, which is a constant. The slope *m* of the liquidus surface depends on the undercooling *ΔT* of BCC and FCC and the element concentration change *ΔC* at the liquid/solid front. The value of *f* is fixed by the alloying elements of FCC and BCC.

The essence of the nanocomposite method of FCC and BCC proposed in this paper is the overall simple combination of two phases. Therefore, it is reasonable to consider it as a binary alloy. We assume that these alloys precipitate in the form of alternating layers at the liquid-solid front, and the precipitation of the microstructure is not only related to the stability of the phase but also involves competitive growth issues. The solidification of the alloy from the molten state to obtain a stable or fine room temperature eutectic microstructure requires Gibbs thermodynamic conditions and supercooling as the thermodynamic driving force for the solidification of the alloy, affecting the thickness *λ* of the lamella. The solid-liquid interface can be subject to composition and temperature conditions, which will lead to changes in the solid phase morphology, solid phase composition, and solid phase fraction.

If precipitation or solidification occurs in the equilibrium state, the surface energy and interface curvature at the three-phase junction (the point where the two solid phases contact the liquid phase *l*) will inevitably change, resulting in changes in the internal pressure of the solid phase $\emptyset$. At this time, the drastic composition fluctuations of the liquid phase *l* will cause the composition of the solid phase $\emptyset$ to be unbalanced. The interface temperature will deviate from the equilibrium value, and the supercooling *ΔT* will serve as the driving force for the eutectic to solidify and grow in the direction of decreasing Gibbs free energy. Senninger and Voorhees decomposed the supercooling into the average supercooling of the two phases:

$\bar{\Delta T\left( \varepsilon\right)}^{FCC}=\sum_{i=2}^{N} m_{i}^{FCC}\left( C_{i}^{l}-{\bar{C_{i}^{l}}}^{FCC} \right)+\frac{2\Gamma_{{FCC}/l}\sin\left( \left| \theta_{FCC} \right| \right)}{f_{FCC}\lambda}$ (S22)

$\bar{\Delta T\left( \varepsilon\right)}^{BCC}=\sum_{i=2}^{N} m_{i}^{BCC}\left( C_{i}^{l}-{\bar{C_{i}^{l}}}^{BCC} \right)+\frac{2\Gamma_{{BCC}/l}\sin\left( \left| \theta_{BCC} \right| \right)}{f_{BCC}\lambda}$ (S23)

If a stable layered structure is to be obtained by solidification under the condition of satisfying Gibbs free energy, the two solid phases should maintain a supercooling condition equilibrium under a constant growth rate *v* and a standard temperature gradient, that is, $\bar{\Delta T\left( \varepsilon\right)}^{FCC}=\bar{\Delta T\left( \varepsilon\right)}^{FCC}$. Under the same temperature conditions at the interface of the two solid phases, the solid-liquid angle *θ* is related to the concentration *∆C*. In the equation $\bar{\Delta T\left( \varepsilon\right)}^{FCC}=\bar{\Delta T\left( \varepsilon\right)}^{FCC}$, the concentration variation range *∆C* is the only condition that needs to be determined. Due to the conservation of matter between the solid and liquid phases, this means that the average concentration of element *i* in the solid phases FCC and BCC ($\bar{C_{i}^{FCC}}$ and $\bar{C_{i}^{BCC}}$) is $f_{FCC}\bar{C_{i}^{FCC}}+f_{BCC}\bar{C_{i}^{BCC}}=-f_{l}C_{i}^{l}$. As solidification proceeds, the liquid phase and concentration gradually decrease, and the back end of the equation approaches 0. Then we get that the concentrations of the two phases are inversely proportional to the volume fractions (Equation S24).

$\frac{f_{FCC}}{f_{BCC}}=-\frac{\bar{C_{i}^{BCC}}}{\bar{C_{i}^{FCC}}}$ (S24)

Therefore, the concentration change at the liquid and solid fronts is also determined by the chemical composition of the two phases in the alloy, which is consistent with the original idea of combining CoCrFeNi-FCC and NiAl-BCC that we designed. It can be predicted that under the conditions of supercooling, growth kinetics, and energy stability, the two-phase alloy we designed can still maintain a high degree of bonding even at the high cooling rate of additive manufacturing and be stably stored at room temperature.

Supplementary Text 5 Strain distribution

**Figure S6** noted that the two samples also have a macroscopic strain field relief effect similar to AM-Nano, such as A1 and B1 lines, although this is not significant. In the intermediate process of straining, plastic flow occurs, but no significant strain concentration is caused under the function of strain concentration relief. The layered heterogeneous structure, as their common structural feature, may provide assistance for the relief of the strain field. In the late stage of plastic deformation, increasingly severe local strain concentration is observed, which is the main cause of material failure.

Plastic flow and strain evolution on smooth metal surfaces tend to be polygonal, point, or circular. The classic theory proposed by Kirsch in 1898 solved the stress/strain concentration problem of plates with circular holes under uniaxial tensile load ^[29]^. We cited the Kirsch method (Eq. S25) calculated the area of the strain influence range through DM3 software, and then equivalent the strain to a circle. The diameter d of the equivalent strain circle can be easily calculated, which is shown in **Table S3**. Due to the instability of strain, the Kirsch rule is combined with the Taylor expansion ^[30],^ ^[31], [32]^ series to obtain Eq. S26. The calculated equivalent strain concentration factor is shown in **Fig. 2I** and **fig. S7**.

$K_{t}=1+2\frac{d}{D}$ S25

$K_{t}=1+2\frac{d}{D}-\frac{3}{2}\left( \frac{d}{D} \right)^{2}+\frac{1}{2}\left( \frac{d}{D} \right)^{3}$ S26

where *K_t_* is the strain concentration factor, *d* is the diameter of the equivalent strain influence range, and *D* is the width of the plate, i.e. the width of the tensile sample.

Supplementary Text 6 Description of tensile static-toughness

The J-integral test method is widely used to evaluate the toughness of metals. Standard specimens required include compact tension (CT) and single edge notch bend (SENB) specimens. These specimens are usually pre-fractured to create a notch that simulates an initial defect, which is a cumbersome experimental process. Tensile static-toughness is the energy a material can absorb without fracturing, calculated as the area under the engineering stress-strain curve ^[33,34]^. Unlike fracture toughness and impact toughness, it is proportional to the product of ultimate tensile strength and uniform elongation (Eq. S27), allowing for direct estimation ^[35]^. This is commonly used in the field of advanced high-strength steels as a standard for assessing overall mechanical performance and strain-hardening ability ^[36]^.

Static-toughness (GPa‧%) = ultimate tensile strength (GPa) × uniform elongation (%) (S27)

Supplementary Text 7 DFT calculation of phase boundary shear resistance

Referring to the FCC and BCC lattice structures obtained by HRTEM experiments of AM-Nano samples (**fig. S9**), the calculation space is expanded in a periodic manner, and the unit cell junction of FCC and BCC is used as the phase boundary. The local calculation ability of DFT calculation in a small space is utilized to load shear stress in the shear direction perpendicular to the phase boundary. Pure FCC shearing causes layer-by-layer slip plane activation (**fig. S10**), resulting in energy release and stress relief effects. Due to the small number of slip systems, the atomic plane of pure BCC shear is difficult to activate in the early process (**fig. S11**), and the shear stress is higher. When the accumulated energy exceeds the barrier, the half-atomic plane is misaligned and dislocations are generated. Combined with the advantages of BCC and FCC, the AM-Nano sample containing the phase interface is combined to exert the continuous energy dissipation function of low-strain complete atomic plane slip and high-strain half-atomic plane misalignment. The shear effects in the horizontal and vertical directions are similar, indicating the phase stability and structural performance stability of the material at a microscale.


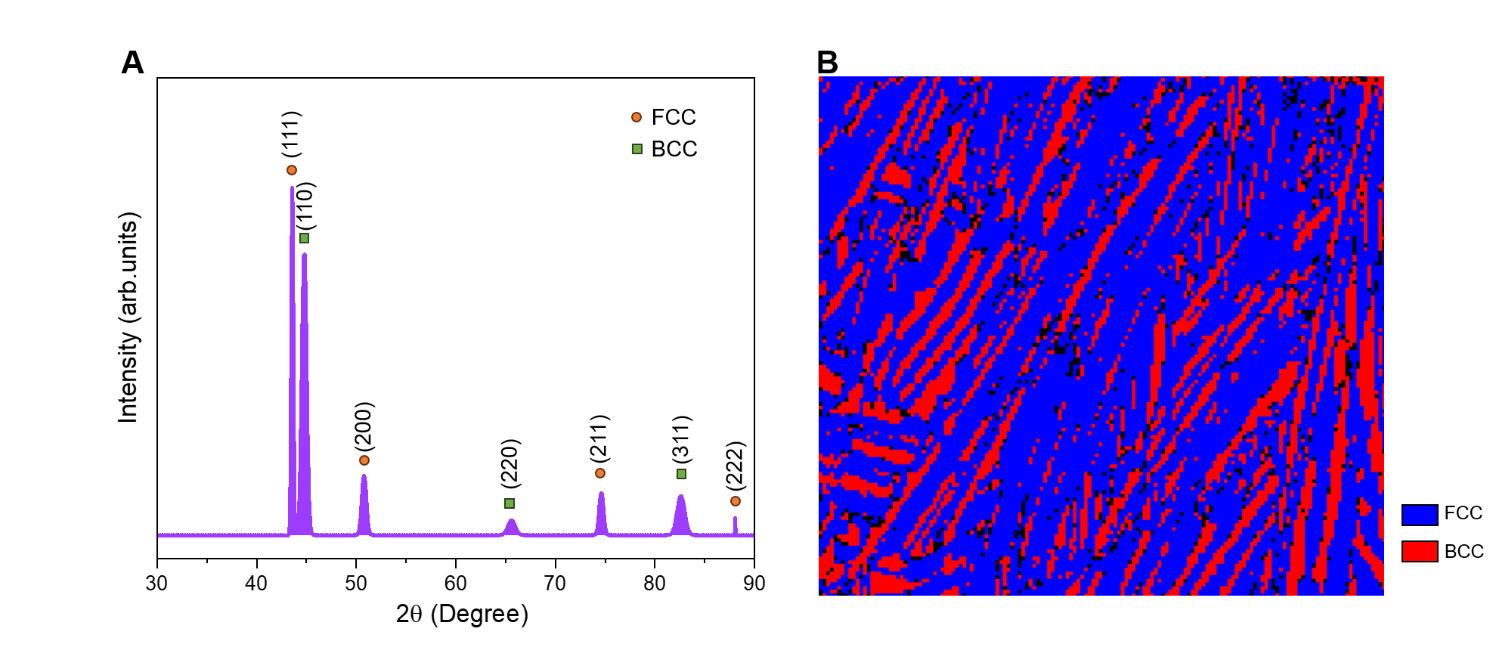


Figure S1. Calibration of XRD diffraction results of AM-Nano samples.

**
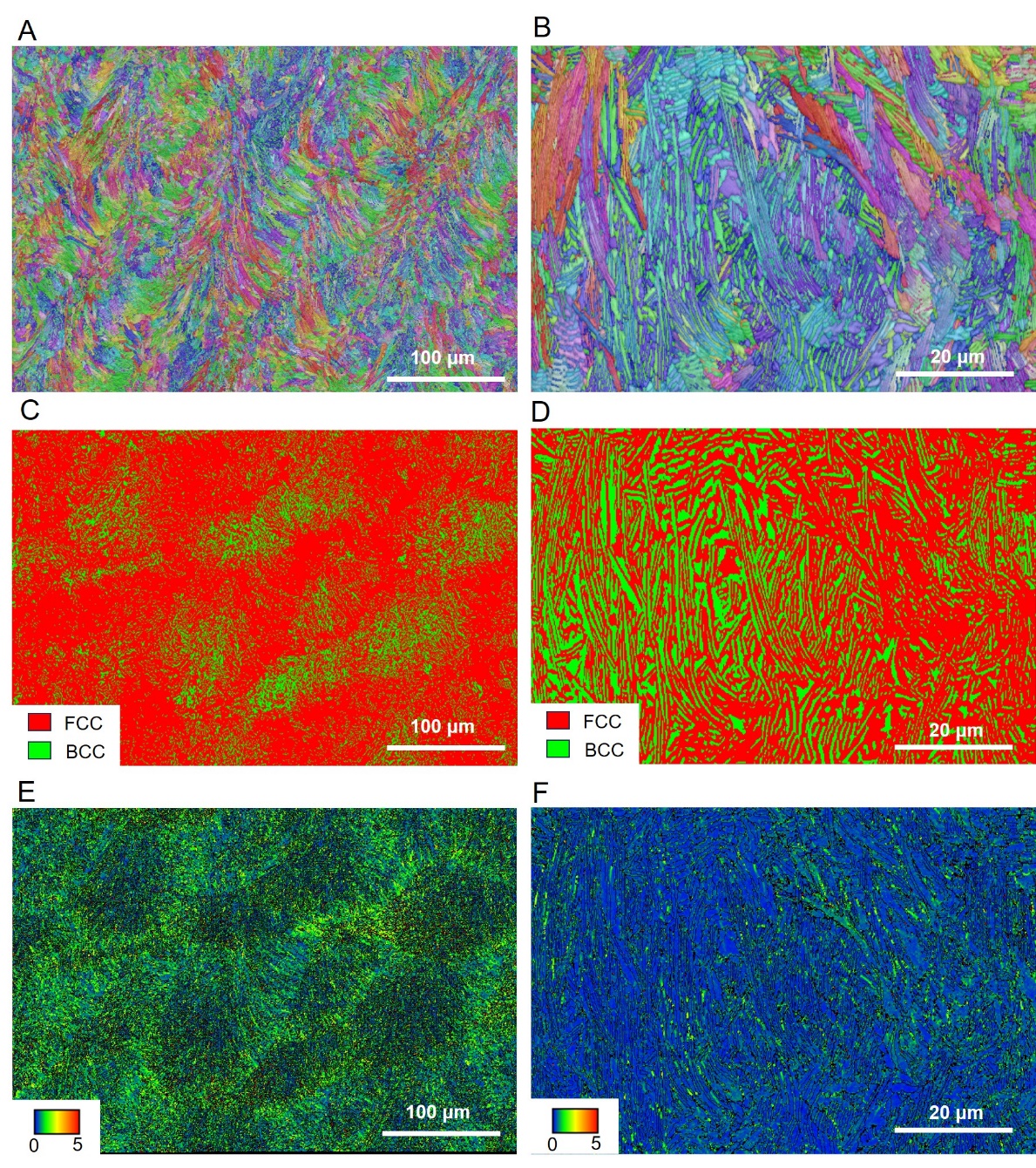
**

**Figure S2. EBSD analysis of AM-Nano samples.** (**A**) Relative general morphology IPF map. (**B**) Local magnified IPF. (**C, D**) Phase distribution maps corresponding to (**A, B**). (E, F) KAM map for (A, B).


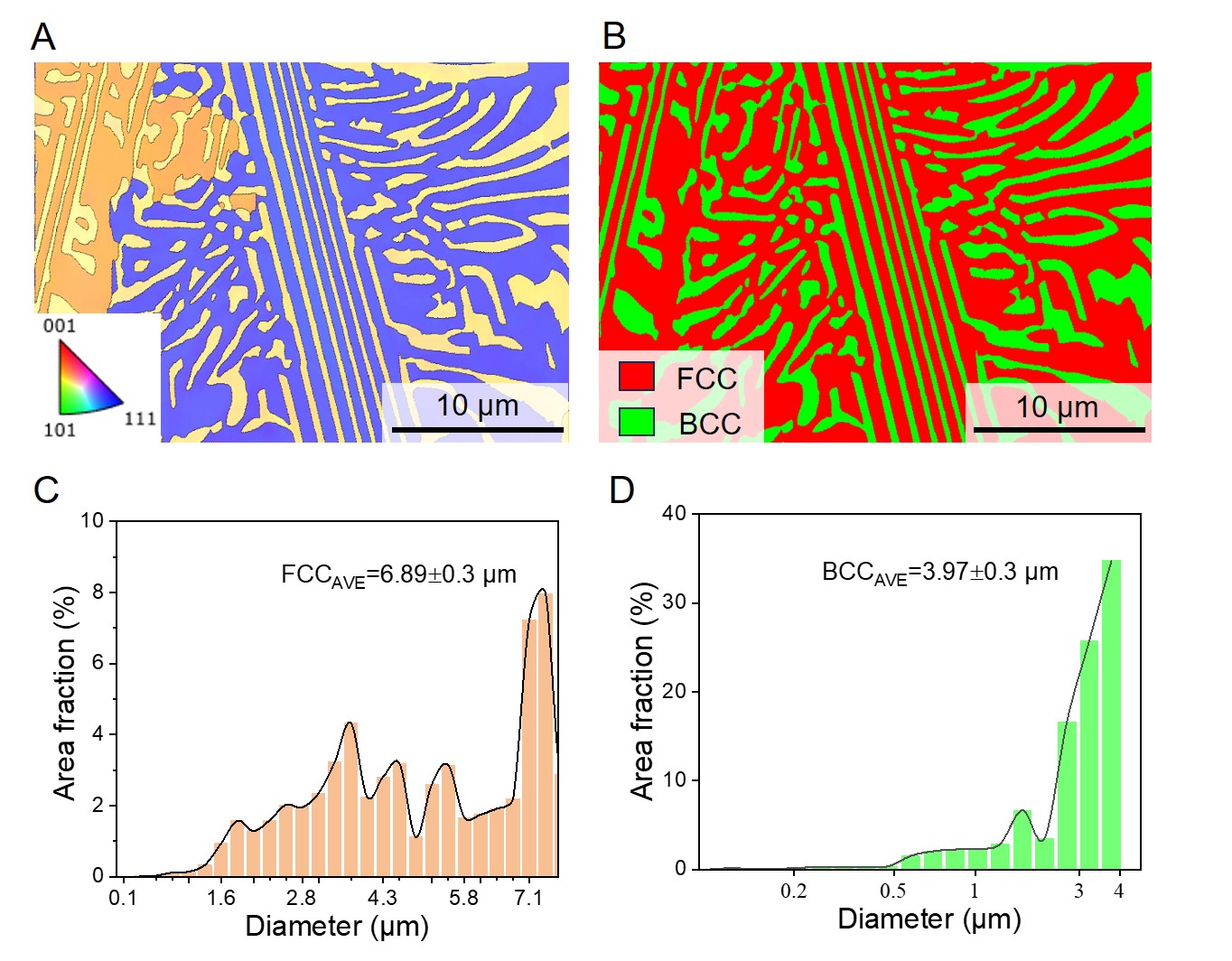


**Figure S3. Cast AS-Coarse EBSD microstructure. (A) IPF. (B) Phase map. (C) FCC size distribution. (D) BCC size distribution.**


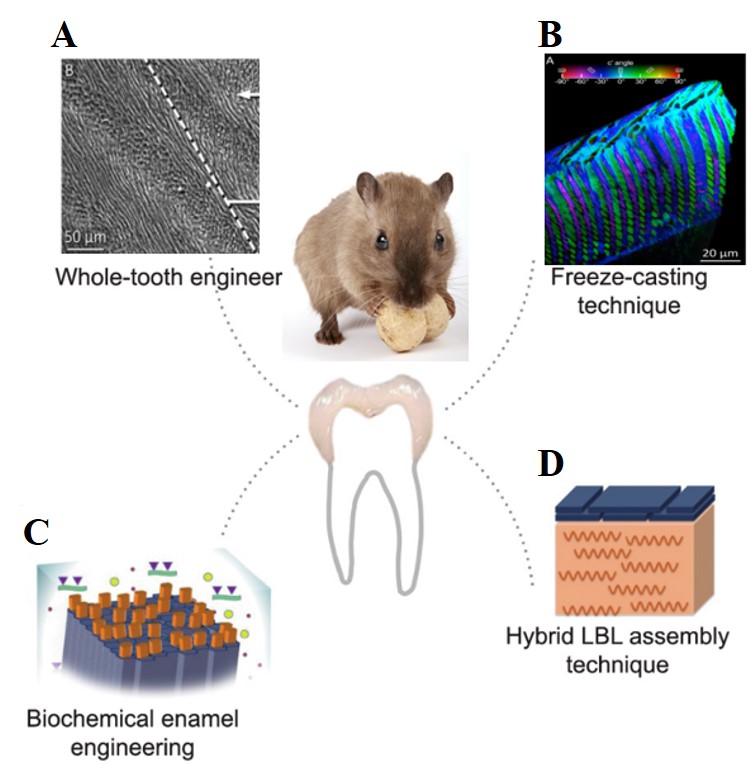


Figure S4. Design concept of biomimetic engineering of mouse tooth enamel (adapted from reference ^[37]^). (A) Mice tooth enamel microstructure ^[38]^. (B) Freeze-casting technique ^[38]^. (C) Biochemical enamel engineering ^[39]^. (D) Hybrid LBL assembly technique ^[39]^.


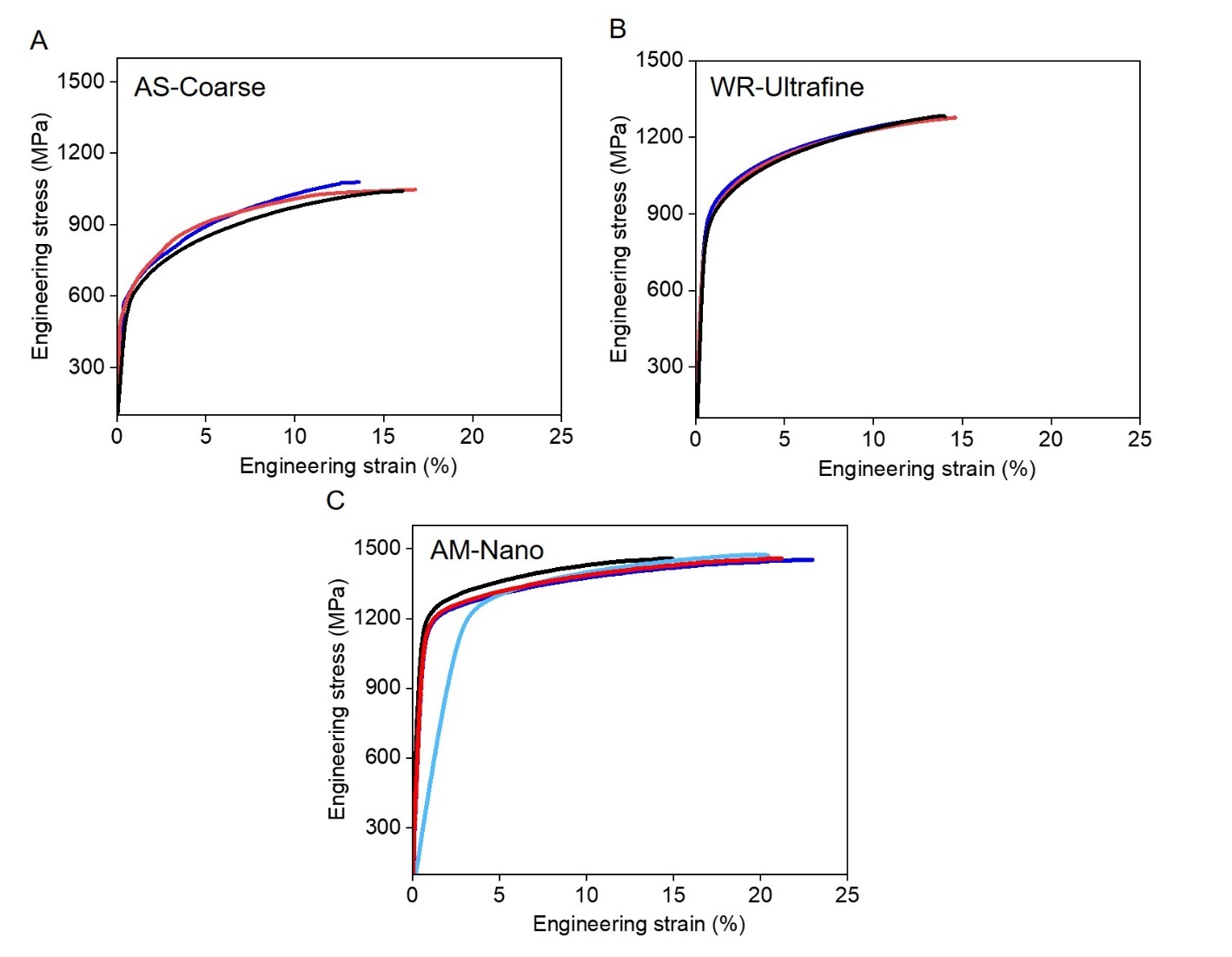


Figure S5. The tensile curves of the three samples were repeated at least three times. (A) AS-Coarse. (B) WR-Ultrafine. (C) AM-Nano.


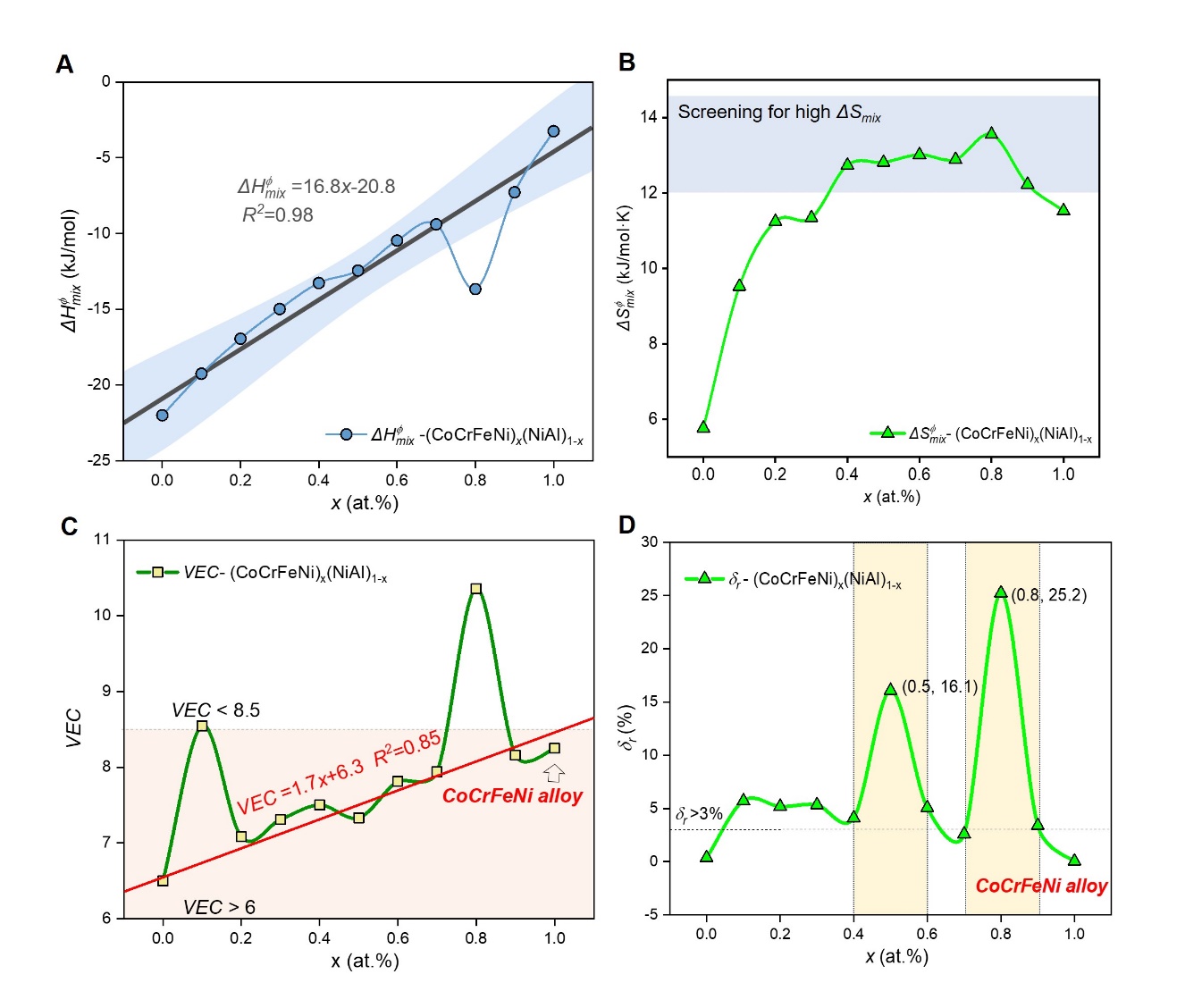


**Figure S6. Variation of** $\boldsymbol{\Delta}\boldsymbol{H}_{\boldsymbol{mix}}^{\boldsymbol{\emptyset}}$**, configurational mixing entropy** $\boldsymbol{\Delta S}_{\boldsymbol{mix}}^{\boldsymbol{\emptyset}}$**, valence electron concentration *VEC* and atomic size difference *δ_r_* of (CoCrFeNi)*_x_*(NiAl)*_1-x_* with the concentration *x* of CoCrFeNi.** (**A)** Variation of mixing enthalpy $\Delta H_{mix}^{\emptyset}$ and linear fitting law. (**B)** Variation of configurational mixing entropy ${\Delta S}_{mix}^{\emptyset}$. (**C, D)** Screening of MAPEs components based on valence electron concentration *VEC* and atomic size difference *δ_r_*.


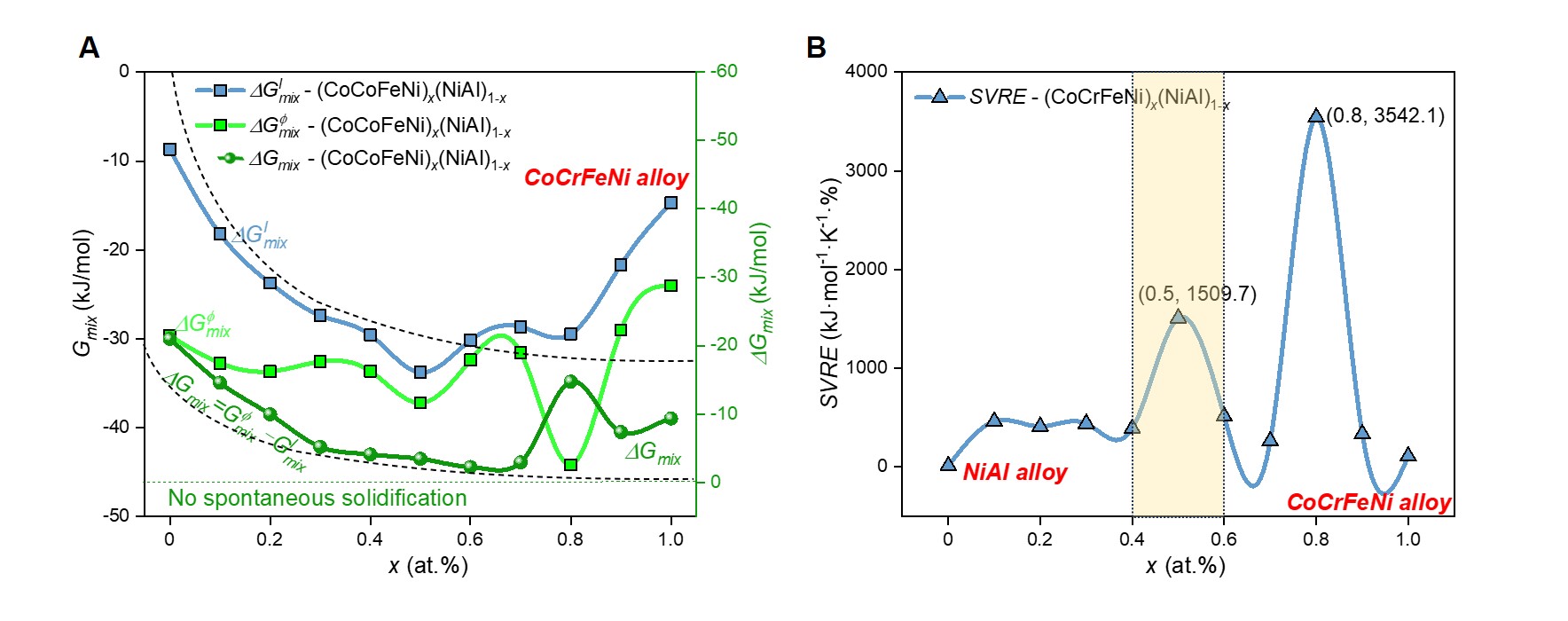


**Figure S7. Calculated Gibbs free energy and SVRE.** (**A)** The liquid Δ*G^l^_mix_* and solid Gibbs free energy ${\Delta G}_{mix}^{\boldsymbol{\emptyset}}$ of (CoCrFeNi)*_x_*(NiTi)_1-_*_x_* alloys at equilibrium position changes with the CrCoFeNi concentration *x*, and (**B)** *SVRE* is calculated to screen the alloys.


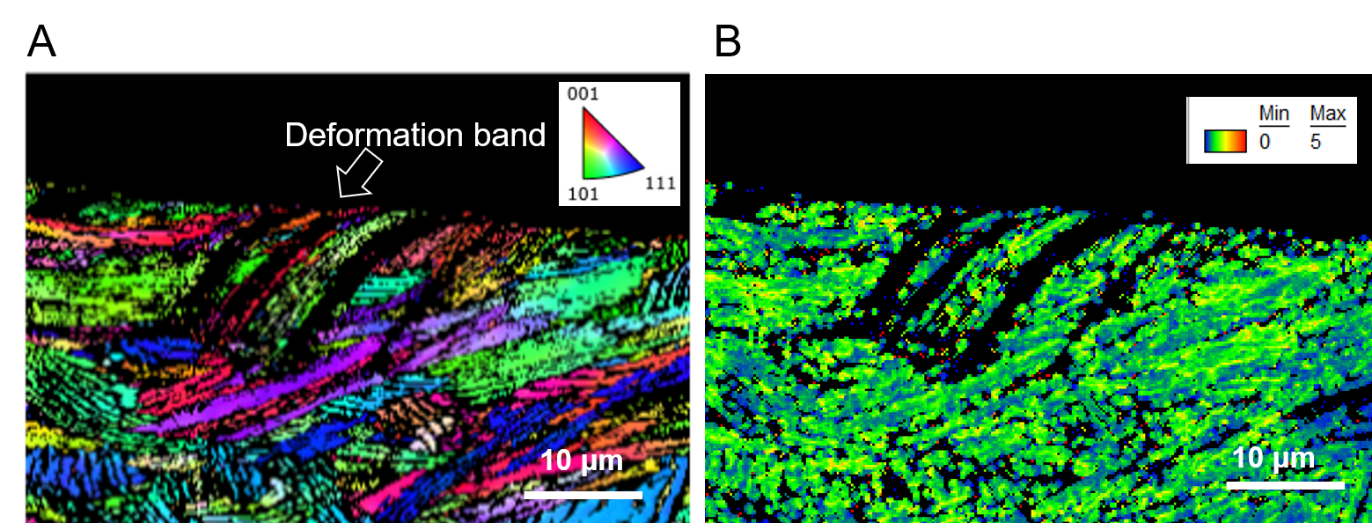


**Figure S8. Deformation band formed on the free surface during in-situ tensile testing. (A)** IPF map. **(B)** KAM map.


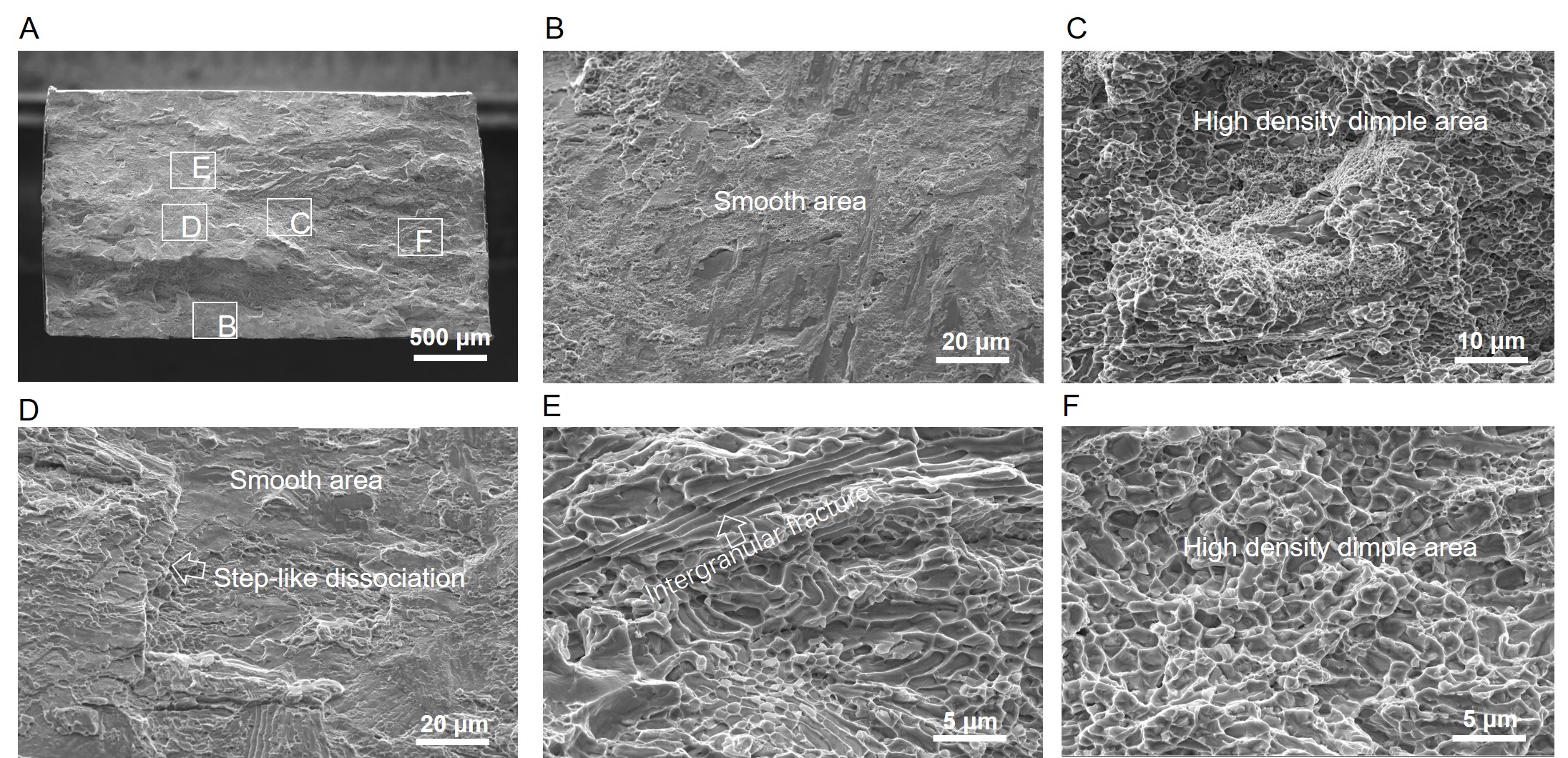


**Figure S9. Fracture analysis of AM-Nano samples after fracture recovery.** (**A**) Macroscopic tensile fracture morphology. (**B**) Smooth area and (**C**) high-density dimple area. (**D**) Step-shaped and (**E**) intergranular fracture characteristics in the smooth area. (**F**) Further magnification of the high-density dimple area.


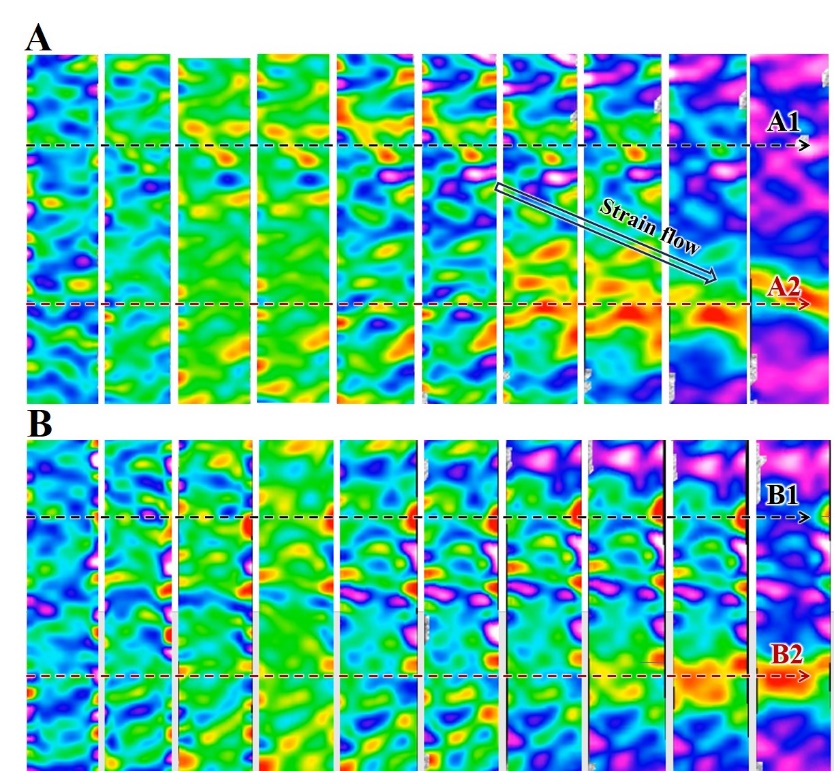


Figure S10. Strain evolution during stretching identified by DIC technique. (A) AS-Coarse and (B) WR-Ultrafine.


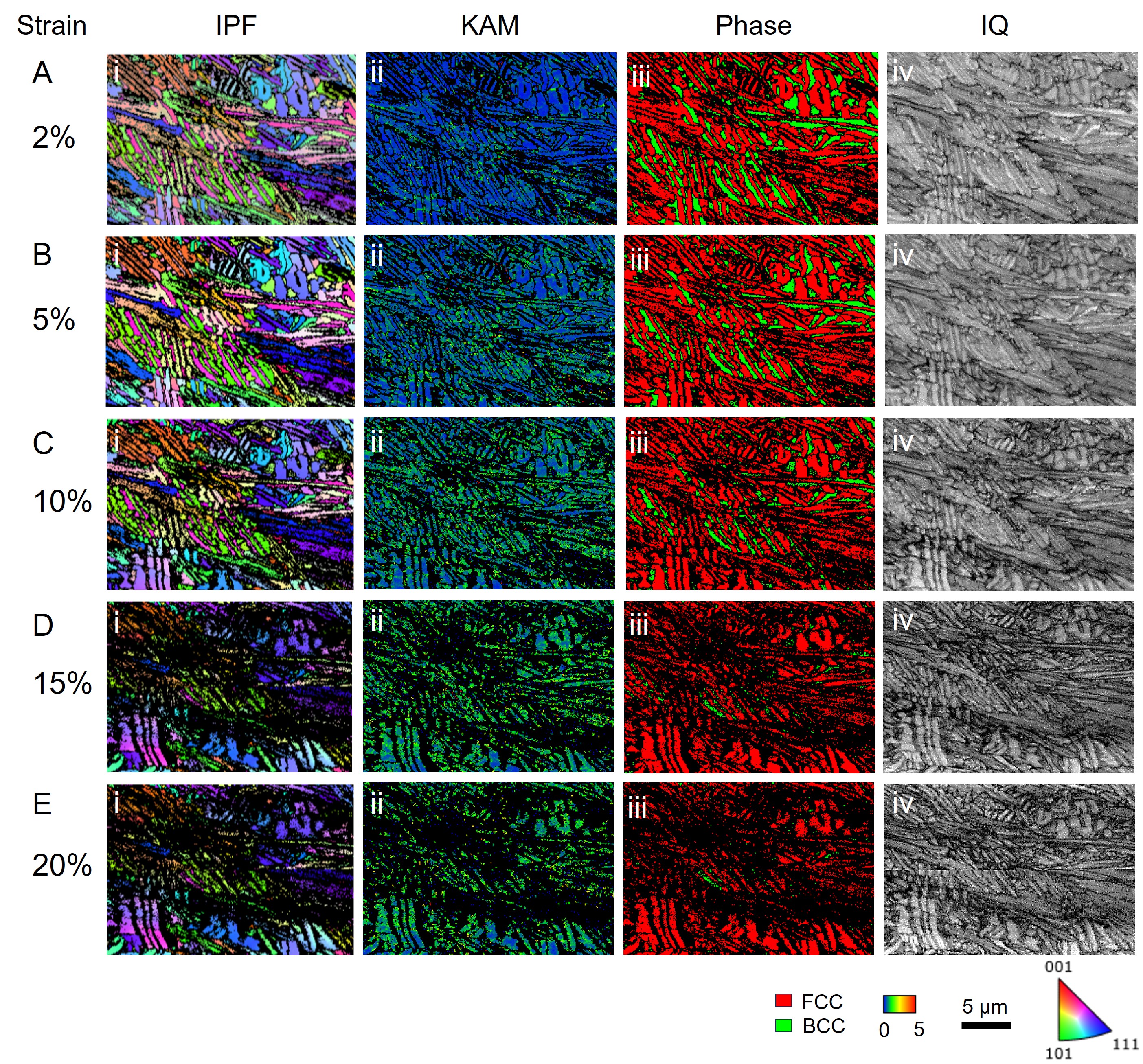


**Figure S11. KAM distribution of AM-Nano sample during in-situ tensile EBSD process. (A-E) Structural evolution process of strain 2-20%. Contains (i-iv) IPF, KAM, Phase and IQ.**


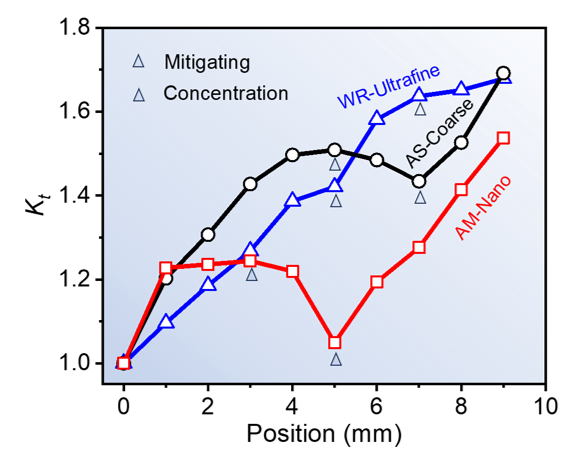


Figure S12. Strain evolution during stretching identified by DIC technique.


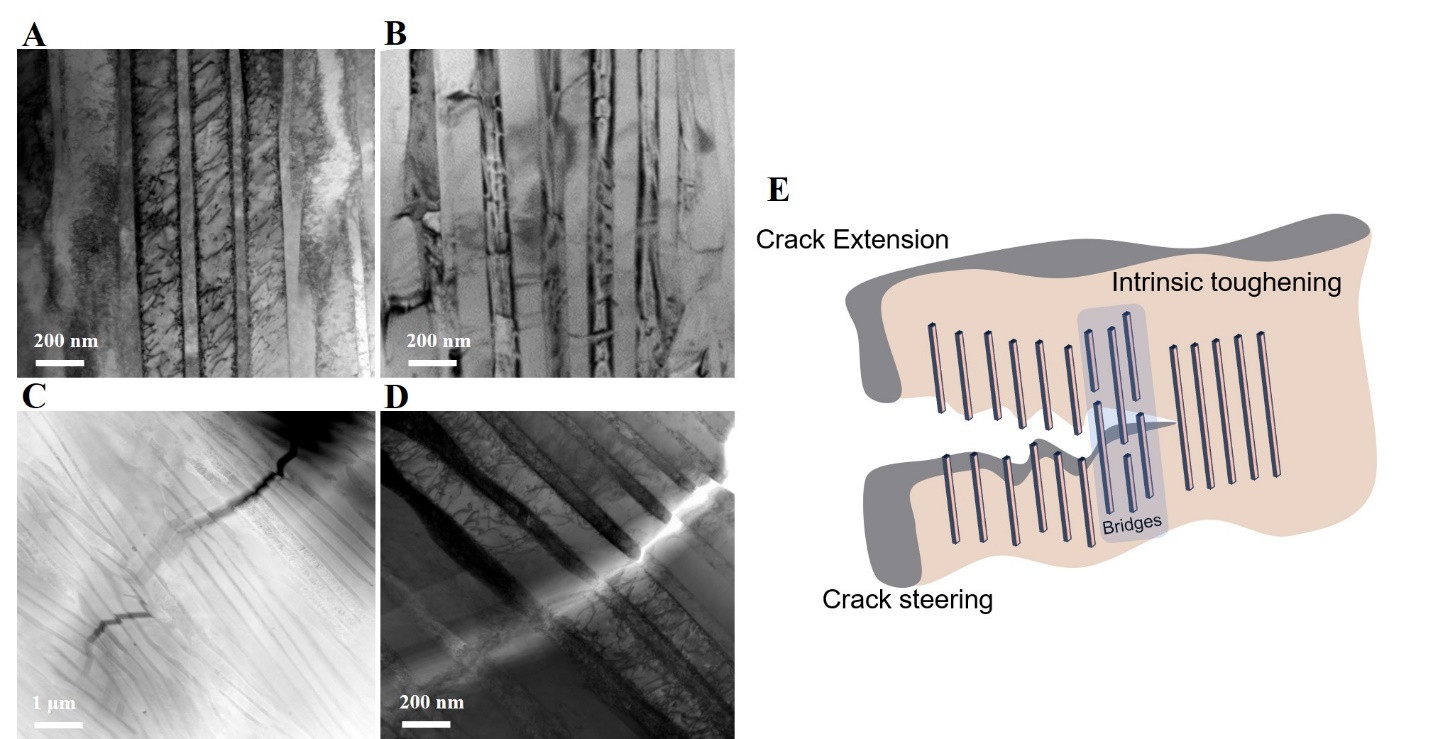


**Figure S13. Dislocation states and lamellar fracture modes in the microstructure of AM-Nano.** (**A)** Accumulation of early dislocations did not cause serious morphological changes in the FCC/BCC interface. (**B)** The number of microcracks in brittle BCC is significantly higher than that in FCC, which is related to the energy absorption effect of FCC and the interface. (**C)** Tortuous propagation route of the crack. (**C)** Coarse crack breaks through the limit under high stress. (**E)** Schematic diagram of crack growth in microstructure.


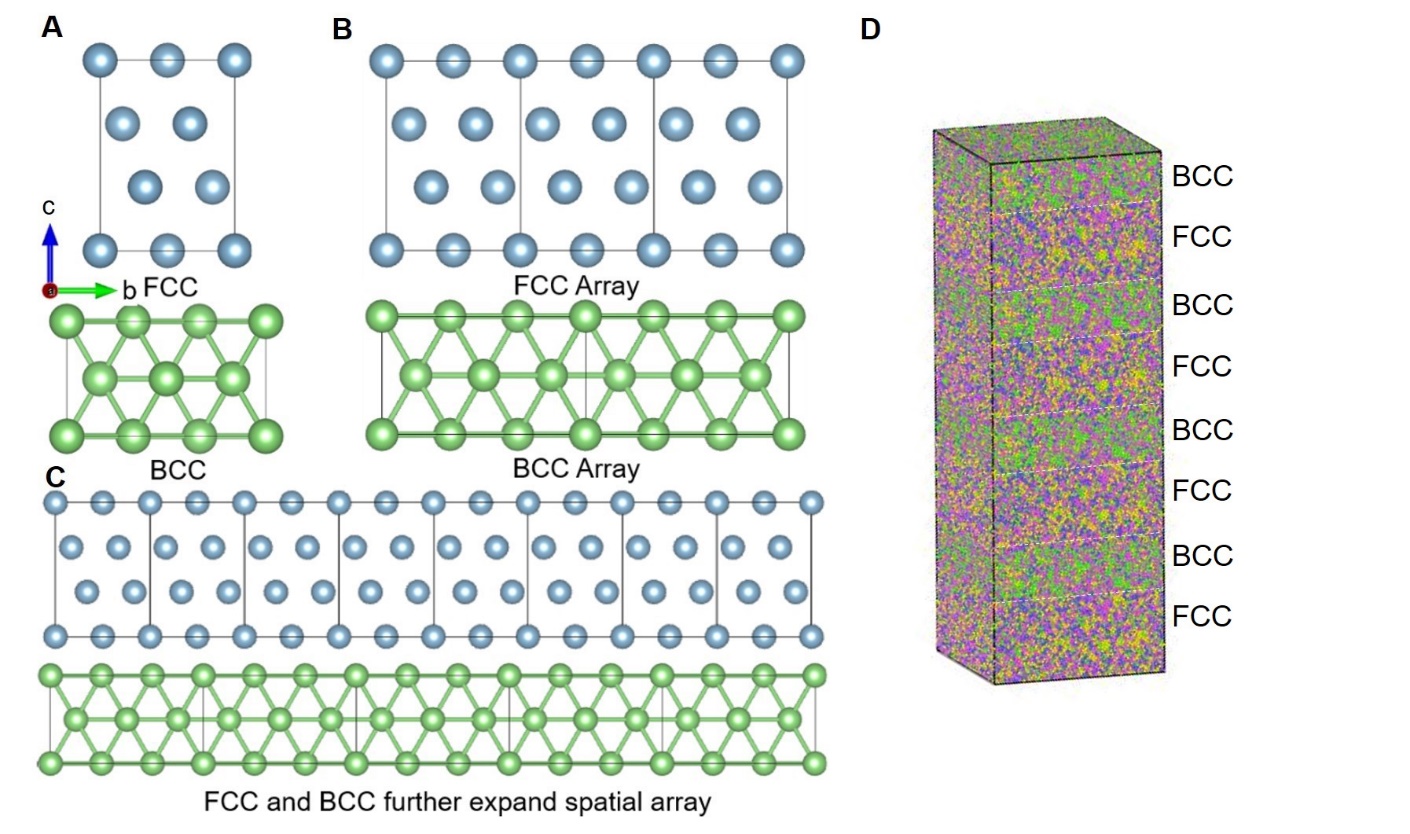


**Figure S14. AM-Nano lattice structure model established by DFT and MD method. (A)** The basic lattice structure of AM-Nano matrix, FCC, and BCC**. (B), (C)** Basic lattice structure is extended to space. (**D**) MD initial model.


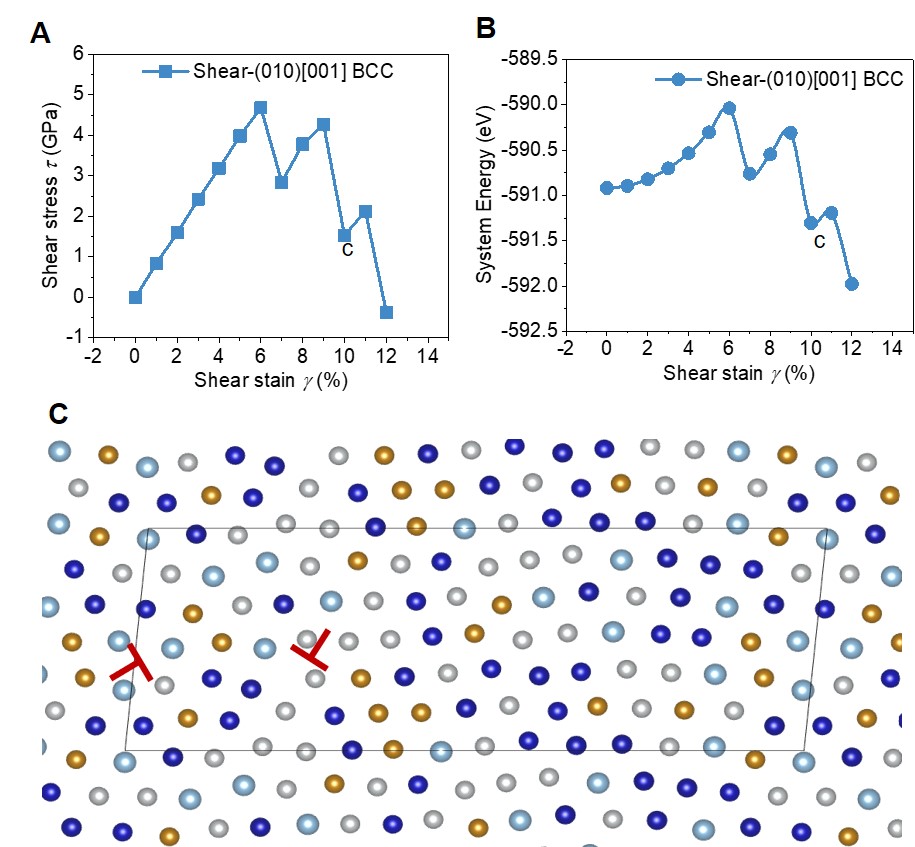


**Figure S15. Local shear exploration of the BCC matrix and the evolution of shear stress and system energy with shear strain. (A), (B)** Relationship between shear stress and system energy and shear strain. (**C)** As the strain progresses, half-atomic planes are generated, inducing the initiation of dislocations. Stress relief and energy release phenomena are found in **(A), and (B)**.


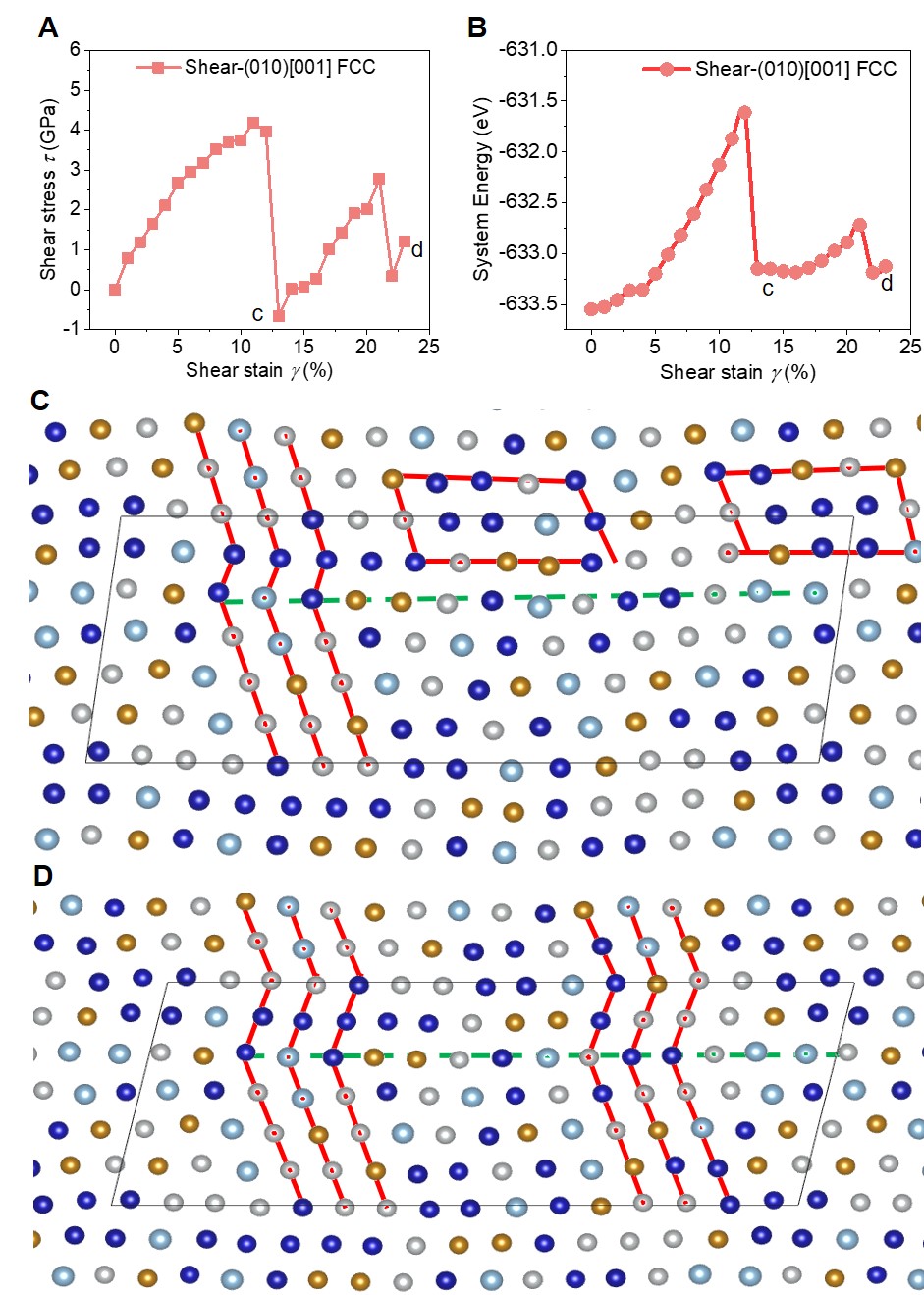


**Figure S16. Local shear exploration of the FCC matrix and the evolution of shear stress and system energy with shear strain. (A), (B)** Relationship between shear stress and system energy and shear strain. (**C), (D)** As the strain progresses, the atomic planes slip layer by layer, causing continuous stress relief and energy release.

**
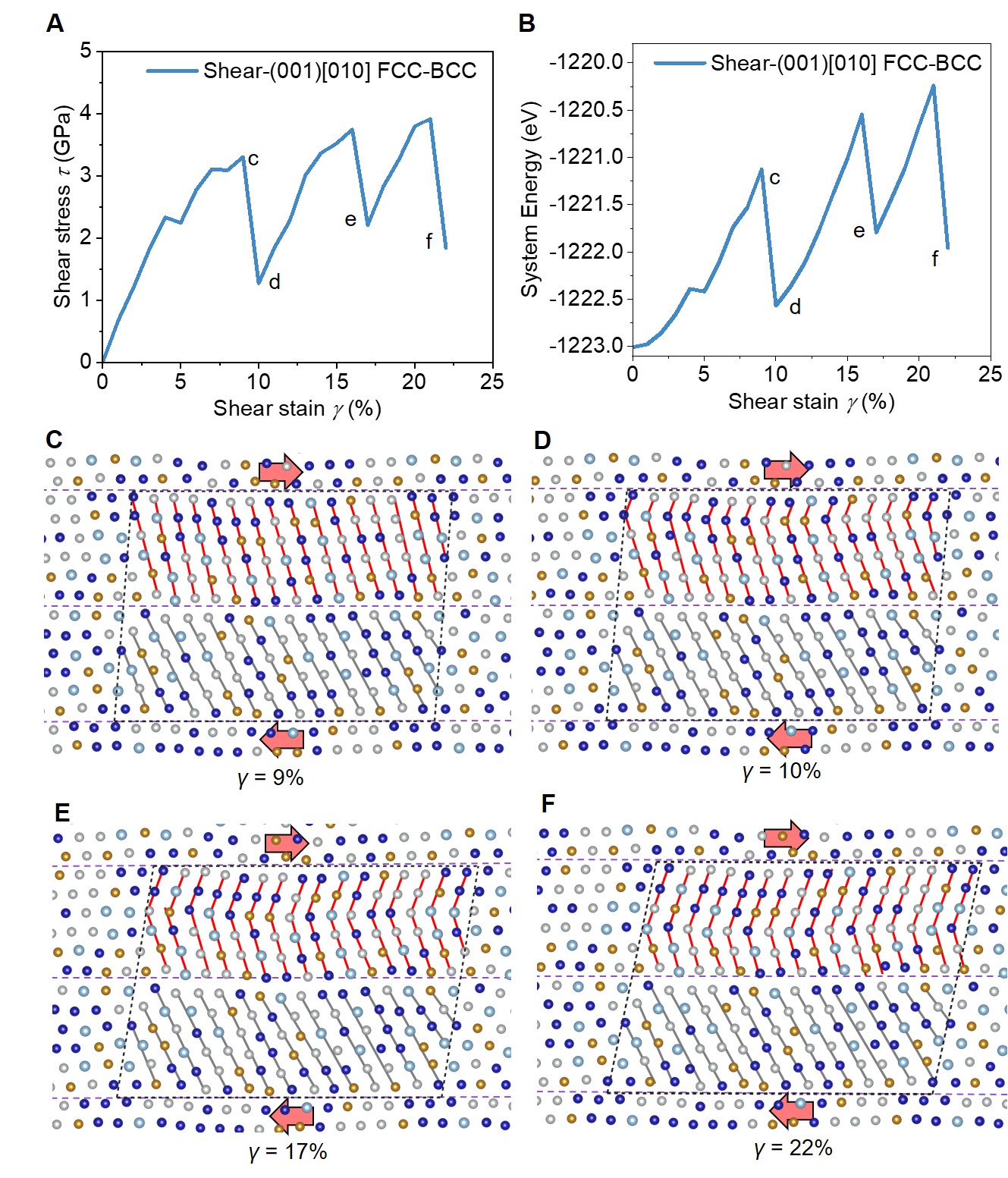
**

**Figure S17. The stress-state and deformation process along the phase boundary direction. (A)** Shear stress-strain curve. **(B)** System energy-strain curve. **(C)** At 9% strain, the system accumulates a high level of energy but still maintains lattice stability. When the shear reaches 10% (**D)**, to accommodate plastic deformation, the FCC atomic planes begin to slip in the direction parallel to the strain. From the 17% (**E)** and 22% (**F)** strain stages, it is evident that stress relief and energy dissipation are mainly caused by the activation of atomic planes.

Table S1. Information on the enthalpy of mixing between equimolar elements (kJ/mol).

| Elements | *Co* | *Cr* | *Ni* | *Ti* | *Al* | *Fe* | *V* | *Mg* |
| --- | --- | --- | --- | --- | --- | --- | --- | --- |
| *Co* | 0 | -4 | 0 | -28 | -19 | -1 | -14 | 3 |
| *Cr* |  | 0 | -7 | -7 | -10 | -1 | -14 | 24 |
| *Ni* |  |  | 0 | -35 | -22 | -2 | -18 | -4 |
| *Ti* |  |  |  | 0 | -30 | -17 | -2 | - |
| *Al* |  |  |  |  | 0 | -11 | -16 | -38 |
| *Fe* |  |  |  |  |  | 0 | -7 | - |
| *V* |  |  |  |  |  |  | 0 | - |
| *Mg* |  |  |  |  |  |  |  | 0 |

Table S2. Basic physical parameters of pure alloying elements.

| Parameters | *Co* | *Cr* | *Ni* | *Ti* | *Al* | *Fe* | *V* | *Mg* |
| --- | --- | --- | --- | --- | --- | --- | --- | --- |
| *VEC* | 9 | 6 | 10 | 4 | 3 | 8 | 5 | 2 |
| *r (pm)* | 125 | 128 | 124 | 147 | 143 | 126 | 134 | 160 |
| *T (K)* | 1768.15 | 2180.15 | 1728.15 | 1941 | 933.15 | 1538 | - | 923 |
| $\boldsymbol{G}_{\boldsymbol{mix}}^{\boldsymbol{l}}$ *(kJ/mol)* | -2.5 | -3.4 | -5.1 | -1.1 | -3.2 | -3.7 | - | -3 |

**Table S3. Strain concentration parameters are calculated and counted based on equivalent strain thinking.**

| ***Locals*** | ***0*** | ***1*** | ***2*** | ***3*** | ***4*** | ***5*** | ***6*** | ***7*** | ***8*** | ***9*** |
| --- | --- | --- | --- | --- | --- | --- | --- | --- | --- | --- |
| ***WR-Ultrafine*** | | | | | | | | | | |
| ***d_A1_*** | 0 | 0.39 | 0.50 | 0.40 | 0.33 | 0.32 | 0.52 | 0.50 | 0.20 | 0 |
| ***d_A2_*** | 0 | 0.10 | 0.20 | 0.30 | 0.46 | 0.51 | 0.78 | 0.89 | 0.92 | 0.98 |
| ***A1-K_t_*** | 1.00 | 1.34 | 1.41 | 1.35 | 1.29 | 1.28 | 1.43 | 1.41 | 1.19 | 1.00 |
| ***A2-K_t_*** | 1.00 | 1.10 | 1.19 | 1.27 | 1.39 | 1.42 | 1.58 | 1.64 | 1.65 | 1.68 |
| ***AS-Coarse*** | | | | | | | | | | |
| ***d_B1_*** | 0 | 0.20 | 0.32 | 0.35 | 0.31 | 0.30 | 0.39 | 0.37 | 0.36 | 0.21 |
| ***d_B2_*** | 0 | 0.22 | 0.35 | 0.52 | 0.63 | 0.65 | 0.61 | 0.53 | 0.68 | 1.01 |
| ***B1-K_t_*** | 1.00 | 1.19 | 1.28 | 1.31 | 1.28 | 1.27 | 1.34 | 1.32 | 1.31 | 1.19 |
| ***B2-K_t_*** | 1.00 | 1.19 | 1.28 | 1.31 | 1.27 | 1.27 | 1.34 | 1.32 | 1.31 | 1.19 |
| ***AS-Nano*** | | | | | | | | | | |
| ***d_Circle_*** | 0 | 0.10 | 0.20 | 0.35 | 0.27 | 0.03 | 0.20 | 0.33 | 0.31 | 0.29 |
| ***d_Arrow_*** | 0 | 0.25 | 0.26 | 0.27 | 0.24 | 0.05 | 0.21 | 0.31 | 0.50 | 0.70 |
| ***Circle-K_t_*** | 1.00 | 1.10 | 1.19 | 1.31 | 1.24 | 1.03 | 1.19 | 1.29 | 1.28 | 1.26 |
| ***Arrow-K_t_*** | 1.00 | 1.23 | 1.24 | 1.24 | 1.22 | 1.05 | 1.19 | 1.28 | 1.41 | 1.54 |

**References**

[1] Y.-J. Liang, L. Wang, Y. Wen, B. Cheng, Q. Wu, T. Cao, Q. Xiao, Y. Xue, G. Sha, Y. Wang, Y. Ren, X. Li, L. Wang, F. Wang, H. Cai, *Nature Communications* **2018**, *9*, 4063.

[2] E. P. George, D. Raabe, R. O. Ritchie, *Nature Reviews Materials* **2019**, *4*, 515.

[3] N. T.-C. Nguyen, P. Asghari-Rad, P. Sathiyamoorthi, A. Zargaran, C. S. Lee, H. S. Kim, *Nature Communications* **2020**, *11*, 2736.

[4] F. Khodabakhshi, M. Mohammadi, A. P. Gerlich, *Journal of Materials Science* **2021**, *56*, 15513.

[5] S. J. Sun, Y. Z. Tian, X. H. An, H. R. Lin, J. W. Wang, Z. F. Zhang, *Materials Today Nano* **2018**, *4*, 46.

[6] M. Murayama, J. M. Howe, H. Hidaka, S. Takaki, *Science* **2002**, *295*, 2433.

[7] M. A. Meyers, A. Mishra, D. J. Benson, *Progress in materials science* **2006**, *51*, 427.

[8] Q. Pan, L. Zhang, R. Feng, Q. Lu, K. An, A. C. Chuang, J. D. Poplawsky, P. K. Liaw, L. Lu, *Science* **2021**, *374*, 984.

[9] K. Lu, L. Lu, S. Suresh, *Science* **2009**, *324*, 349.

[10] J. Hu, Y. N. Shi, X. Sauvage, G. Sha, K. Lu, *Science* **2017**, *355*, 1292.

[11] X. Liu, K. Song, Z. Kou, J. Gong, X. Chen, Q. Gao, H. Sun, P. Liu, R. Qu, L. Hu, Z. Zhang, P. Ramasamy, Z. Liu, Z. Zhang, F. Liu, Z. Zhang, J. Eckert, *International Journal of Plasticity* **2024**, *177*, 103992.

[12] T. Li, T. Liu, S. Zhao, Y. Chen, J. Luan, Z. Jiao, R. O. Ritchie, L. Dai, *Nature Communications* **2023**, *14*, 3006.

[13] J. Du, W. Li, Z. Huang, H. Feng, Y. Li, *Journal of Materials Research and Technology* **2024**, *30*, 3268.

[14] D. Gu, X. Shi, R. Poprawe, D. L. Bourell, R. Setchi, J. Zhu, *Science* **2021**, *372*, eabg1487.

[15] P. Shi, R. Li, Y. Li, Y. Wen, Y. Zhong, W. Ren, Z. Shen, T. Zheng, J. Peng, X. Liang, P. Hu, N. Min, Y. Zhang, Y. Ren, P. K. Liaw, D. Raabe, Y.-D. Wang, *Science* **2021**, *373*, 912.

[16] Y. Chen, Y. Zheng, Y. Zhou, W. Zhang, W. Li, W. She, J. Liu, C. Miao, *Nature Communications* **2023**, *14*, 3438.

[17] D. Nepal, S. Kang, K. M. Adstedt, K. Kanhaiya, M. R. Bockstaller, L. C. Brinson, M. J. Buehler, P. V. Coveney, K. Dayal, J. A. El-Awady, L. C. Henderson, D. L. Kaplan, S. Keten, N. A. Kotov, G. C. Schatz, S. Vignolini, F. Vollrath, Y. Wang, B. I. Yakobson, V. V. Tsukruk, H. Heinz, *Nature Materials* **2023**, *22*, 18.

[18] L. Jin, M. Zhang, L. Shang, L. Liu, M. Li, Y. Ao, *Composites Science and Technology* **2020**, *200*, 108382.

[19] K. Jackson, J. Hunt, in *Dynamics of Curved Fronts*, Elsevier, **1988**, pp. 363–376.

[20] W. Hume-Rothery, G. Mabbott W., K. Channel Evans, *Philosophical Transactions of the Royal Society of London. Series A, Containing Papers of a Mathematical or Physical Character* **1934**, *233*, 1.

[21] Z. An, A. Li, S. Mao, T. Yang, L. Zhu, R. Wang, Z. Wu, B. Zhang, R. Shao, C. Jiang, B. Cao, C. Shi, Y. Ren, C. Liu, H. Long, J. Zhang, W. Li, F. He, L. Sun, J. Zhao, L. Yang, X. Zhou, X. Wei, Y. Chen, Z. Lu, F. Ren, C.-T. Liu, Z. Zhang, X. Han, *Nature* **2024**, *625*, 697.

[22] H. Li, H. Zong, S. Li, S. Jin, Y. Chen, M. J. Cabral, B. Chen, Q. Huang, Y. Chen, Y. Ren, *Nature* **2022**, *604*, 273.

[23] Z. An, T. Yang, C. Shi, S. Mao, L. Wang, A. Li, W. Li, X. Xue, M. Sun, Y. Bai, Y. He, F. Ren, Z. Lu, M. Yan, Y. Ren, C.-T. Liu, Z. Zhang, X. Han, *National Science Review* **2024**, *11*, nwae026.

[24] A. Miedema, *Journal of the less common metals* **1973**, *32*, 117.

[25] Y. Zhang, Y. J. Zhou, J. P. Lin, G. L. Chen, P. K. Liaw, *Advanced Engineering Materials* **2008**, *10*, 534.

[26] B. Chanda, J. Das, *Journal of Alloys and Compounds* **2019**, *798*, 167.

[27] K. A. Jackson, J. D. Hunt, in *Dynamics of Curved Fronts* (Ed.: P. Pelcé), Academic Press, San Diego, **1988**, pp. 363–376.

[28] O. Senninger, P. W. Voorhees, *Acta Materialia* **2016**, *116*, 308.

[29] E. G. Kirsch, *Zeitshrift des Vereines deutscher Ingenieure* **1898**, *42*, 797.

[30] D. V. Kubair, B. Bhanu-Chandar, *International Journal of Mechanical Sciences* **2008**, *50*, 732.

[31] A. M. Dotsenko, A. N. Polyakov, *Strength of Materials* **1989**, *21*, 1699.

[32] M. Pourahmadi, *The American Mathematical Monthly* **1984**, *91*, 303.

[33] Q. Wang, B. Sang, J. Q. Ren, C. Xin, Y. H. Zhang, Q. Gao, W. F. Liu, Z. L. Ning, J. T. Yu, X. F. Lu, *International Journal of Plasticity* **2024**, *174*, 103920.

[34] Z. N. Yang, F. C. Zhang, F. C. Liu, Z. G. Yan, Y. Y. Xiao, *Materials & Design* **2012**, *40*, 400.

[35] H. Li, H. Zong, S. Li, S. Jin, Y. Chen, M. J. Cabral, B. Chen, Q. Huang, Y. Chen, Y. Ren, K. Yu, S. Han, X. Ding, G. Sha, J. Lian, X. Liao, E. Ma, J. Sun, *Nature* **2022**, *604*, 273.

[36] C. Hu, C. P. Huang, Y. X. Liu, A. Perlade, K. Y. Zhu, M. X. Huang, *Acta Materialia* **2023**, *245*, 118629.

[37] L. Zhang, Y. Zhang, T. Yu, L. Peng, Q. Sun, B. Han, *Engineering* **2022**, *14*, 113.

[38] C. A. Stifler, J. E. Jakes, J. D. North, D. R. Green, J. C. Weaver, P. U. P. A. Gilbert, *Acta Biomaterialia* **2021**, *120*, 124.

[39] L. Zhang, Y. Zhang, T. Yu, L. Peng, Q. Sun, B. Han, *Engineering* **2022**, *14*, 113.
